# Voxel-accurate MRI-microscopy Correlation Enables AI-powered Prediction of Brain Disease States

**DOI:** 10.7150/thno.125235

**Published:** 2026-03-17

**Authors:** Julian Schroers, Yvonne Yang, Ekin Reyhan, Nirosan Sivapalan, Einar Ismail-Zade, Alina Heuer, Jonas G. Scheck, Atefeh Pourkhalili Langeroudi, Obada T. Alhalabi, Tara Moghiseh, Manuel Fischer, David Batra, Johann Jende, Felix Sahm, Bogdana Suchorska, Franziska Maria Ippen, Dieter Henrik Heiland, Matthia A. Karreman, Franz L. Ricklefs, Michael O. Breckwoldt, Felix T. Kurz, Varun Venkataramani

**Affiliations:** 1 Neurology Clinic, National Center for Tumor Diseases and European Center for Neurooncology (EZN), Heidelberg University Hospital, Heidelberg, Germany.; 2 Division of Radiology, German Cancer Research Center (DKFZ), Heidelberg, Germany.; 3 Clinical Cooperation Unit Neurooncology, German Cancer Consortium (DKTK), German Cancer Research Center (DKFZ), Heidelberg, Germany.; 4 Department of Neuroradiology, Heidelberg University Hospital, Heidelberg, Germany.; 5 Department of Neurosurgery, Heidelberg University Hospital, Heidelberg, Germany.; 6 Department of Neuropathology, Heidelberg University, Heidelberg, Germany.; 7 German Cancer Consortium (DKTK), Germany.; 8 Translational Neurosurgery, Friedrich-Alexander University Erlangen Nuremberg, Erlangen, Germany.; 9 Department of Neurosurgery, University Hospital Erlangen, Friedrich-Alexander University Erlangen Nuremberg, Erlangen, Germany.; 10 Department of Neurological Surgery, Northwestern University Feinberg School of Medicine, Chicago, IL, USA.; 11 Department of Neurosurgery, University Medical Center Hamburg-Eppendorf (UKE), Hamburg, Germany.; 12 Clinical Cooperation Unit Neuroimmunology and Brain Tumor Immunology, German Cancer Research Center (DKFZ), Heidelberg, Germany.; 13 Division of Neuroradiology, Geneva University Hospitals, Geneva, Switzerland.

**Keywords:** MRI, intravital light microscopy, co-registration, glioblastoma, deep learning algorithms.

## Abstract

**Rationale:**

Magnetic resonance imaging (MRI) is essential for visualizing the healthy and diseased brain, yet the cellular basis of MRI signal and how it changes over time remain poorly understood.

**Methods:**

Here, we present **BRIDGE** (**B**rain **R**adiological **I**maging with **D**eep-learning based **G**round-Truth **E**xploration), a platform integrating *in vivo* MRI with *in vivo* two-photon (2P) and *ex vivo* super-resolution microscopy using a multi-step, iterative co-registration pipeline. It enables *in vivo*, longitudinal and voxel-precise mapping of MRI signals to their biological ground truth for the first time. The registered overlay reveals the cellular and anatomical origins of MRI signals and enables training of convolutional neural networks to enhance the effective resolution of MRI.

**Results:**

Using BRIDGE, we identified a microenvironmental vessel biomarker for early metastatic colonization in patient-derived xenograft models of breast cancer brain metastasis. In particular, we found that distinct T2*-weighted hypointense lesions correspond to reduced blood flow and erythrostasis in perimetastatic capillaries. In glioma, longitudinal intravital studies further demonstrated direct correlations between non-vasogenic T2-weighted signal changes and patient-dependent tumor growth dynamics.

**Conclusions:**

Taken together, BRIDGE advances radiological interpretation by establishing a microscopic ground truth for MRI signatures over time, enabling deep learning-based predictive histology and providing cellular level insights into tumor microenvironment with direct clinical imaging implications.

## Introduction

Magnetic resonance imaging (MRI) is an important radiological technology to visualize structural and anatomical abnormalities and disease processes 1. Core sequences, such as T1-weighted (T1w), T2-weighted (T2w) and T2*-weighted (T2*w), are essential in diagnosing and monitoring neurological conditions, particularly those involving brain vasculature and tumors 2-4. Radiological assessments rely on qualitative evaluations of visible abnormalities, introducing subjective variability due to the expertise of practitioners 4-7. The emergence of radiomics, multimodal data analysis and quantitative MRI introduced a quantitative description of imaging signatures 8-10. More recently, artificial neural networks have been applied to tasks such as predictive modeling and synthetic sequence generation, offering new tools for clinical assessment 11, 12.

However, current AI-assisted predictions rely on manual human segmentation, which can be variable and lacks robust validation 8, 13. Therefore, an accurate correlation of microscopic cellular ground truth with MRI signal is needed.

Recent studies have started to simultaneously acquire MRI and microscopy images 14-20. However, existing pipelines for correlative imaging using two-photon (2P) microscopy and MRI either did not directly overlay microscopy and MRI modalities 14, 15 or were not able to provide a voxel-accurate multimodal registration 16. While light-sheet microscopy enables the investigation of whole mouse brain volumes, it is limited to static, single-time-point measurements at microscopic resolution 17, 21-23. Therefore, the need for longitudinal studies capable of tracking changes in single voxels over time with 3D spatial information and precise image co-registration remains unmet. So far, it remains unclear whether microscopy-based ground truth co-registered with MRI could enable AI-driven prediction of cellular structures directly from MRI data.

To address these limitations, we developed BRIDGE (**B**rain **R**adiological **I**maging with **D**eep-learning based **G**round-Truth **E**xploration), a pipeline enabling comprehensive voxel-to-voxel accurate co-registration between multimodal *in vivo* MRI, intravital 2P microscopy and *ex vivo* light and super-resolution microscopy in the healthy brain as well as in patient-derived brain tumor models, including glioblastoma and brain metastases. With BRIDGE, we acquired 126 correlative datasets, comprising patient-derived xenograft models and tissue resected from human patients. To enhance structural detection and subsequent automatic segmentation, BRIDGE utilizes microscopy data as ground truth to train MR images with convolutional neural networks 24, 25. Moreover, BRIDGE is suitable for understanding the biological processes involved in neurological diseases and their effects on MRI signal. Previously in glioblastoma, attempts have been made to attribute biological significance to T2 signal: Preceding research has demonstrated that non-local tumor progression is associated with T2-hyperintense, non-contrast-enhancing tumor regions 26. This has led to the hypothesis that T2-hyperintensity could be an early indicator of glioma progression. However, it is still controversial how exactly these temporal changes are related to disease progression and if they could guide clinical decisions 27. In particular, the differentiation between edema and tumor progression in T2-positive areas is still challenging 28, 29. These observations lack histopathological correlation and biological ground truth 30. Therefore, BRIDGE enables correlation of glioblastoma dynamics with MRI signal changes. Lastly, we provide an approach to translate these findings to the human disease.

## Results

### Multimodal and longitudinal co-registration bridges MRI and *in* and *ex vivo* light microscopy resolution

To enable voxel-accurate co-registration between intravital two-photon (2P) microscopy and magnetic resonance imaging (MRI), we developed the BRIDGE pipeline consisting of repetitive longitudinal correlative 2P and 9.4T high-resolution MRI acquisitions.

**Figure 1:**
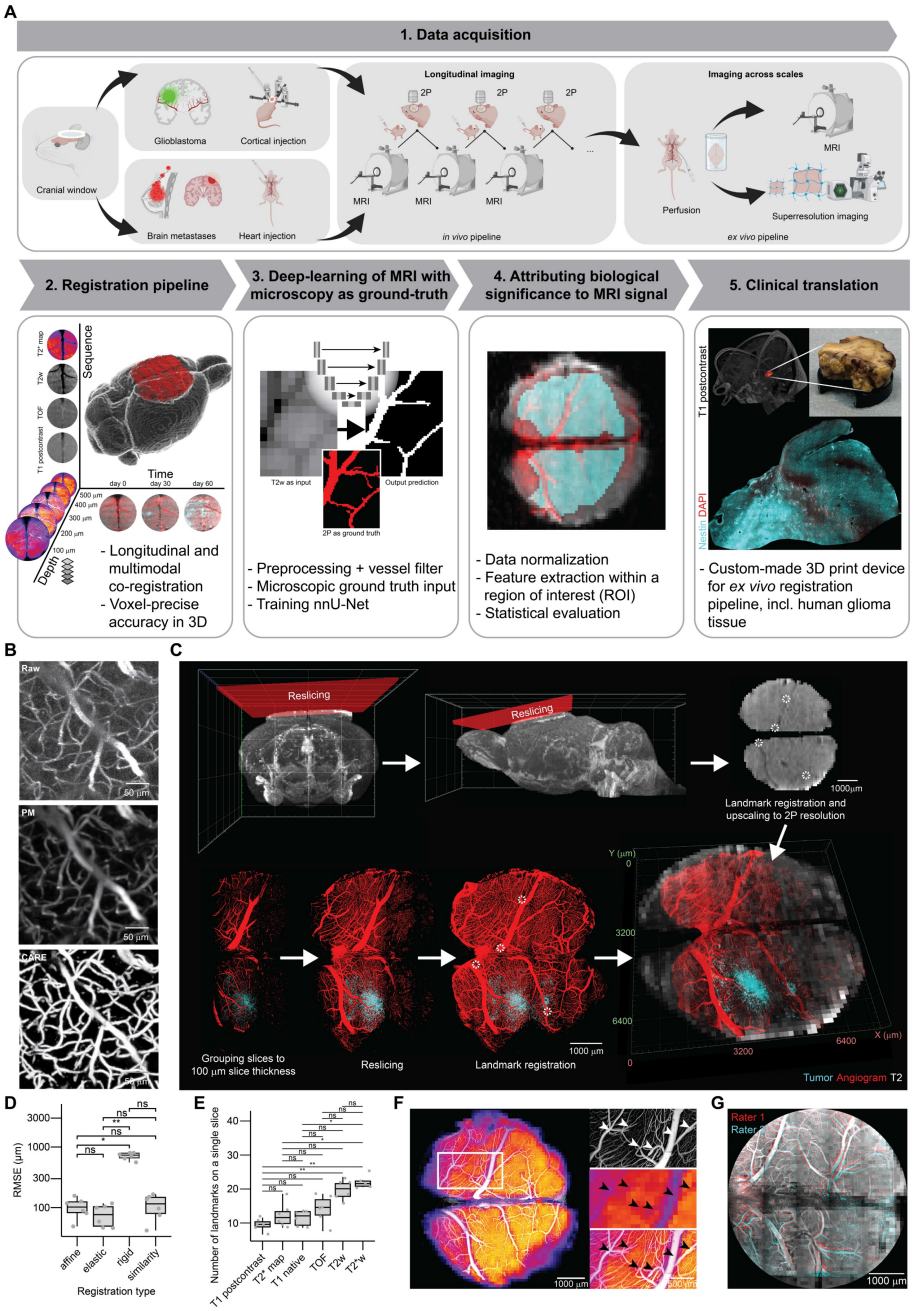
** Overview and methodology of the BRIDGE pipeline. A,** The BRIDGE pipeline consists of 5 major steps: Data acquisition, co-registration pipeline, deep-learning based image restoration, attribution of biological meaning to MRI signal and clinical translation. **B,** Content-aware restoration (CARE) of 2P microscopy and subsequent interactive machine learning segmentation. **C,** Multistep and iterative registration workflow including reslicing MRI and 2P images, rescaling, and elastic 3D landmark registration using blood vessel landmarks. **D,** RMSE comparison across registration modes. Median with interquartile range (n = 6 datasets per registration mode, Kruskal-Wallis test followed by Dunn-Bonferroni post-hoc test). **E,** Number of identified landmarks across MRI sequences. Median with interquartile range (n = 6 datasets per sequence, Kruskal-Wallis test followed by Dunn-Bonferroni post-hoc test). **F,** Co-registered T2* map and 2P-based angiogram demonstrating the precision of the pipeline. Arrowheads indicate registration accuracy of small vessels**. G,** Example of inter-rater evaluation.

Custom-made teflon rings instead of conventional titanium rings were used for cranial window surgery in mice to minimize MRI artifacts and enable accurate co-registration 15 **(Figure S1A)**. Postoperative edema was visible in MRI beneath the cranial window until two weeks after surgery **(Figure S1B)**. Subsequently, control datasets of mice without pathology were recorded. In patient-derived and brain-tropic xenograft models of glioblastoma and brain metastases, dynamics of tumor growth and vessel architecture changes were observed in repetitive, longitudinal MRI and 2P acquisitions **(step 1 in Figure 1A)**.

After postprocessing and co-registration, multidimensional output was generated containing information in multiple MRI sequences (T1 native, T1 post-contrast, TOF, T2w, T2*w and T2* map), temporal resolution provided by longitudinal measurements and increased imaging resolution and depth achieved through 2P microscopy which allows for improved tissue penetration **(step 2 in Figure 1A, Movie S1)**.

Using a convolutional neural network for image segmentation 24 **(step 3 in Figure 1A)**, BRIDGE enhances structural detection using microscopy data as a ground truth to train MR images. To attribute biological significance to MRI signal, voxel-to-voxel-registered data, data normalization, ROI selection and statistical evaluation were used for downstream analyses and interpretation **(step 4 in Figure 1A)**. Finally, we demonstrated how *in vivo* MRI of glioblastoma patients could be directly correlated with confocal and super-resolution microscopy 31-36 **(step 5 in Figure 1A)**.

Acquiring cortex-wide fluorescent angiograms is crucial for bridging macroscopic MRI resolution with the microscopic resolution, as uniquely identifiable vessels and vessel branching points serve as co-registration landmarks. However, broad spatial coverage requires rapid imaging as anesthesia time of mice is limited 37, which can compromise image resolution and signal-to-noise-ratio (SNR). Here, we incorporated deep-learning-based content-aware restoration 25 and interactive machine learning 38, 39 to enhance both SNR and resolution across the spatial imaging areas between 6.30x10^9^ μm^3^ and 2.11x10^10^ μm^3^ **(Figure 1B)**.

For cross-modality co-registration, preprocessed 2P and MR images underwent signal intensity normalization and multiple registration steps in BRIDGE including reslicing multimodal imaging data parallel to the chronic window and an elastic, vessel bifurcation landmark-based, iterative co-registration method **(Figures 1C, S1C-D)**.

To achieve the most accurate co-registration possible, we evaluated the registration error of four different registration algorithms that included affine, elastic, rigid and similarity registration. Elastic registration showed a tendency towards being the most precise co-registration **(Figure 1D)**. Furthermore, we assessed different MR sequences for the number of visible anatomical registration landmarks to achieve the smallest possible registration error. We found that both T2w and T2*w sequences 40, 41 had the highest number of identifiable registration points in 3D **(Figures 1E, S1E)**. When correlating 2P images to T2* maps, even small vessels with a diameter down to approximately 40 µm showed correspondence between the angiogram and T2* visible vessels **(Figure 1F, Movie S1)**. The registration accuracy of BRIDGE was validated through inter-rater evaluations, achieving a low average root mean square error (RMSE) of 71.6 µm **(Figure 1G)**.

To push the margins of image resolution, we performed imaging across scales using BRIDGE and different modalities of microscopy and successfully correlated 9.4T MRI to super-resolution Airyscan imaging 42, 43 *in vivo* **(Figure S1F)**. For following quantitative analyses, the resolution of the 2P images was downsampled and matched to the resolution of the MR images (see Materials and Methods). Subsequently, the corresponding voxel intensities were extracted from the registered 2P and MRI data within a region of interest (ROI) **(Figure S1G)**.

### Ground-truth-based deep learning predictions improve the detection of brain vasculature in MRI

To make a precise statement about the correlation between biological ground truth and MRI signal, it is essential to first assess whether the physiological structures depicted in MRI and 2P are continously visible, so that changes can be attributed to pathology. In T2w images, it appears that vascular visibility remains predominantly constant over time **(Figure S2A-B)**. For all visibility categories, the maximum absolute deviation from the reference timepoint was well within a predefined equivalence margin of ±5%. No relevant shifts in vessel visibility were observed between timepoints. The same appears to be true in the other sequences **(Figure S2C)**. To ensure equal contrast agent uptake, we quantified signal intensity changes in the sigmoid sinus on pre- and post-contrast T1w images **(Figure S2D)**. Percent signal enhancement (PSE) 44 was 244.7 ± 44.9 % (mean ± SD, n = 6 datasets), corresponding to a coefficient of variation of 18.3 % **(Figure S2E)**. This low inter-animal variability indicates consistent contrast agent uptake and systemic distribution across animals. To verify equal distribution of the fluorescent dextrans within the brain vasculature, we repeatedly imaged the same mice at weekly intervals. Our hypothesis was that the vascular architecture would remain consistent across sessions due to our standardized injection protocol. Using a custom-written analysis script, we quantified the proportion of different vessel diameters within a mask covering the vascular network **(Figure S3A)**. This allowed us to assess whether the vessel diameter distribution remained stable across repeated measurements. Indeed, our results confirmed that the vascular architecture remained uniformly visible across imaging sessions **(Figure S3B)**, as for all diameter categories the deviations from the reference distribution were within the predefined equivalence margin of ±5%. Even small arterioles remained constantly visible over weeks **(Figure S3C)**.

**Figure 2:**
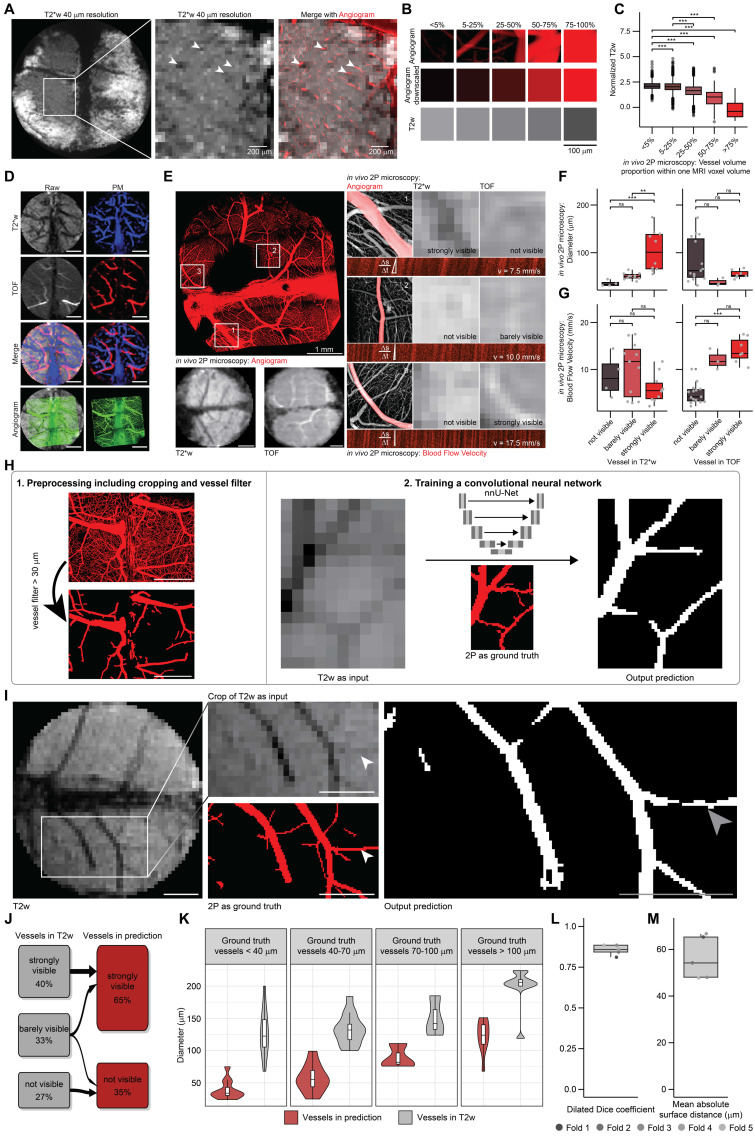
** BRIDGE workflow enables ground-truth based deep learning of MRI. A,** Correlation of *in vivo* high-resolution MRI (T2*w, isotropic 40 µm) with corresponding 2P microscopy angiogram. Arrowheads indicate registration accuracy of small vessels. Scale bars: 200 μm. **B,** Exemplary *in vivo* 2P-correlated MR voxels grouped based on vessel volume proportion. Scale bar: 100 μm. **C,** Vessel volume proportion plotted against normalized T2w signal intensity* in vivo*, presented as median with interquartile range (0-5%, n = 1906 voxels; 5-25%, n = 3334 voxels; 25-50%, n = 801 voxels; 50-75%, n = 196 voxels; >75%, n = 39 voxels; ANOVA with post-hoc Tukey HSD test). **D,** Probability maps of correlated *in vivo* multisequence 2P-MRI, with T2*w (blue), TOF (red), and 2P as ground truth (green). Scale bars: 1 mm.** E,** 2P intravital brain angiogram imaging mapped onto *in vivo* multisequence MRI, with examples of blood flow velocity measurements using line scans in 2P microscopy. Scale bars: 1 mm. **F,** Diameter of vessels depending on visibility across *in vivo* MRI modalities (T2*w, TOF), presented as median with interquartile range (n = 2 mice; for T2*w: not visible, n = 4 vessels; barely visible, n = 11 vessels; visible, n = 4 vessels; strongly visible, n = 6 vessels; for TOF: not visible, n = 15 vessels; barely visible, n = 3 vessels; visible, n = 4 vessels; strongly visible, n = 3 vessels; Kruskal-Wallis test followed by Dunn-Bonferroni post-hoc test). **G,** Blood flow velocity in vessels depending on visibility across* in vivo* MRI modalities (T2*w, TOF). Median with interquartile range (n values same as e, Kruskal-Wallis test followed by Dunn-Bonferroni post-hoc test). **H,** Schematic of the BRIDGE training pipeline including data preprocessing and training of nnU-Net. Data preprocessing contains custom-designed vessel diameter filter. Scale bars: 1 mm. **I,** BRIDGE pipeline uses T2w images as input and 2P microscopy images as ground truth. The output is a prediction of vessels depending on the MR image and the previous training of other datasets. Arrowheads indicate small vessel which was only barely visible in T2w before training. Scale bars: 1 mm. **J,** River plot showing the vessel visibility in the T2w images and in the predictions by the neural network (n = 6 validation datasets, 51 vessels). **K,** Violin plots comparing the vessel diameters in predictions by the neural network and in T2w sequences. Each violin plot shows a different diameter category of vessels in 2P microscopy as ground truth (n = 6 validation datasets, 51 vessels). **L,** Boxplot shows Dilated Dice coefficient between ground truth and prediction. Median with interquartile range (n = 5 folds). **M,** Boxplot shows mean absolute surface distance (MASD) between ground truth and prediction. Median with interquartile range (n = 5 folds).

We applied the BRIDGE framework to analyze the brain vessels and their visibility across different MRI sequences (T2w, T2*w, TOF) 40, 41, 45-47. Voxel information from co-registered 2P and MRI data were extracted for quantitative analyses **(Figure S1G)**. Utilizing a cryogenic coil, higher spatial resolution of 40 µm in 3D T2*w sequences enabled *in vivo* visualization of microvascular details, allowing confident correlation of vessels observed in 2P imaging, down to approximately 20 µm **(Figures 2A, S3D)**.

Voxel data were categorized based on 2P microscopy-derived blood vessel volume per voxel **(Figure 2B)**. Previous studies have noted the role of venous blood vessels in contributing to hypointense voxels in T2w and T2*w imaging 48. Consistent with these findings, our BRIDGE workflow demonstrated a significant inverse correlation between 2P vessel volume density per MRI voxel and T2w intensity **(Figure 2C)**. Subsequently, we assessed the visibility of blood vessels across different MRI sequences. MR-visible arteries and veins were segmented using T2* maps and Time-of-Flight (TOF) sequences 49 **(Figure 2D)**, correlating their MRI characteristics with parameters measured in 2P, such as diameter and blood flow velocity **(Figures 2E, S3E)**. Although TOF signals strongly correlated with blood flow velocity, they did not correlate with vessel diameter. In contrast, T2w and T2*w sequences were effective in delineating vascular anatomy, correlating well with vessel diameter but not flow velocity. Even vessels with smaller diameters than the voxel size were visible in the MR images **(Figures 2A, F-G, S3F)**. The findings support existing knowledge on TOF sequences for visualizing blood flow 50 and provide further evidence through direct comparison with the microscopic ground truth.

In the next step, we leveraged our voxel-accurate co-registration to investigate whether AI-based image restoration with 2P microscopy as ground truth is able to enhance the effective resolution and accuracy of automatic MR segmentation. The aim was to enhance the visibility of vessel architecture at near-microscopic resolution in MRI and to demonstrate the effectiveness of this approach.

Utilizing correlated *in vivo* 2P microscopic information as ground truth **(Figure S4A)**, we used the nnU-Net deep-learning architecture 24 to automatically segment blood vessels in T2w images **(Figure 2H)**. This AI-assisted prediction enhanced the visibility of smaller vessels **(Figure 2I)**. Many vessels that were barely visible in the T2w sequence became clearly visible for human evaluators while a minority of vessels were no longer visible after training **(Figure 2J)**. In each diameter category of ground truth vessels, the estimation of the diameter by the neural network prediction is significantly better than the binarized T2w sequence **(Figure 2K)**.

To assess the training, we performed a 5-fold cross-validation 51 **(Figure S4B)**. The Dilated Dice coefficient 52 was determined to be 0.86 **(Figure 2L)**. The mean absolute surface distance (MASD) 53 is about 54 μm **(Figure 2M)**, which is smaller than the isotropic MRI voxel size of 100 μm. We also performed a neural network training in which the segmentations were randomly flipped horizontally or vertically, in contrast to the MR images, to validate the above procedure. As expected, the results of this training were poor and not able to predict vessel structures of the ground truth **(Figure S4C)**. To summarize, this approach is able to reduce human impact on segmentations and improve the visibility of blood vessels and the estimation of the vessel diameter, thereby highlighting the potential of microscopy-trained MRI.

### Real-time correlative MRI and intravital microscopy reveal ground truth of perimetastatic T2*w changes

Previous findings suggested that T2*w hypointensities in brain metastases is caused by unspecific microbleedings 54. Here, we investigated whether our multimodal pipeline is able to define a microscopic ground truth for T2*w hypointensities in a patient-derived xenograft model of brain metastases for breast cancer 55.

**Figure 3:**
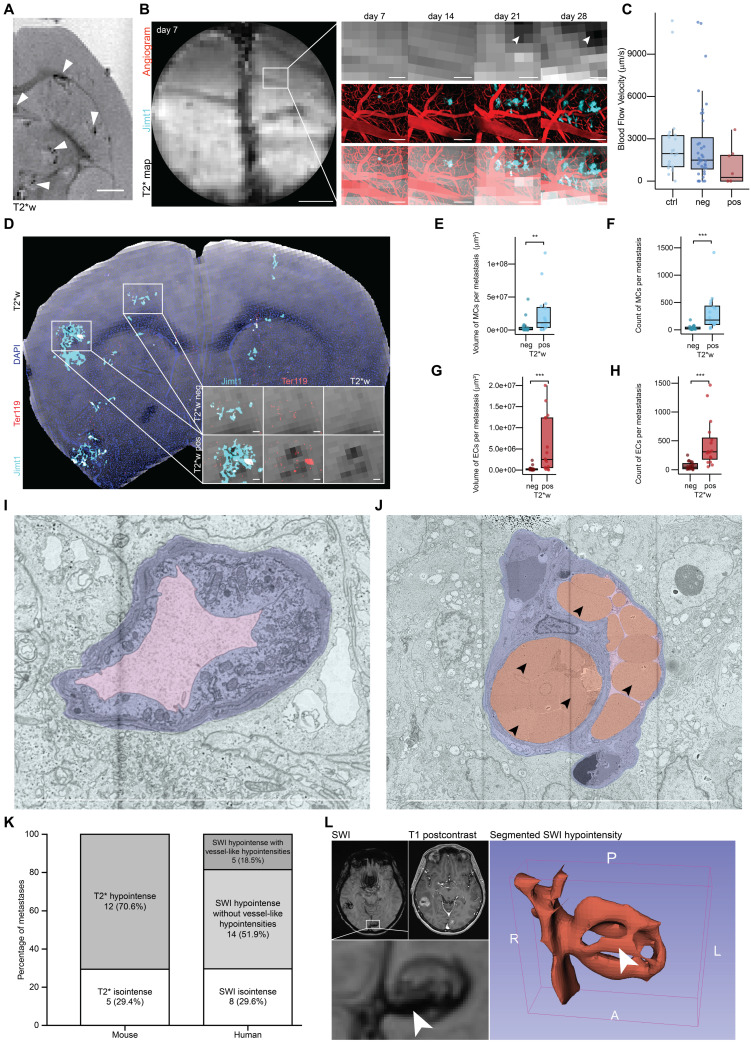
BRIDGE decodes perimetastatic T2*w changes during brain metastastic progression. A, *Ex vivo* MRI screening of breast cancer brain metastasis model JIMT-1 using T2*w imaging. Arrowheads show hypointense changes. Scale bar: 1 mm. **B,** Weekly* in vivo* 2P-MRI correlative imaging of an emerging T2*w hypointense JIMT-1 metastasis (cyan) with angiogram (red) over 28 days. Scale bars: 1 mm (T2* map overview) and 200 µm. **C,** Blood flow velocity measurements of perimetastatic capillaries (JIMT-1 mGFP in cyan) and controls with a diameter <12 μm across different *in vivo* T2* map images. Median with interquartile range (control, n = 22; T2*w negative, n = 36; T2*w positive, n = 8). **D,** *Ex vivo* MRI-LM correlation in a JIMT-1 mGFP (cyan) xenografted mouse brain slice with DAPI (blue) and Ter119 (red) staining, registered onto *ex vivo* T2*w. Insets show T2*-negative and T2*-positive lesions. Scale bars: 100 µm. **E,** Comparison of total volume of metastatic cell clusters (MCs) per metastasis between T2*-negative and T2*-positive metastases *ex vivo*. Median with interquartile range (n = 3 mice; T2*-negative, n = 20; T2*-positive, n = 16; Mann-Whitney U test). **F,** Comparison of the count of MCs per metastasis between T2*-negative and T2*-positive metastases* ex vivo*. Median with interquartile range (n = 3 mice; T2*-negative, n = 20; T2*-positive, n = 16; Mann-Whitney U test). **G,** Comparison of total volume of erythrocyte clusters (EC) per metastasis between T2*-negative and T2*-positive lesions* ex vivo*. Median with interquartile range (n = 3 mice; T2*-negative, n = 20; T2*-positive, n = 16; Mann-Whitney U test). **H,** Comparison of the count of ECs per metastasis between T2*-negative and T2*-positive lesions* ex vivo*. Median with interquartile range (n = 3 mice; T2*-negative, n = 20; T2*-positive, n = 16; Mann-Whitney U test).** I,** Electron microscopy (EM) of perimetastatic capillary showing a vessel with an empty lumen. JIMT-1 (cyan) and perimetastatic capillaries with endothelium (purple) and lumen (pink). Scale bars: 10 µm. **J,** EM of perimetastatic capillary showing a vessel with erythrostasis and a capillary loop formation. Arrowheads indicate erythrocytes. JIMT-1 (cyan) and perimetastatic capillaries with endothelium (purple), erythrocytes (red) and lumen (pink). Scale bars: 10 µm. **K,** Distribution of T2* visibility in breast cancer brain metastases in PDX mouse model (n = 17 metastases in 1 mice) and human MRI (n = 27 metastases in 10 patients). **L,** Segmented perimetastatic SWI hypointensity of a human breast cancer brain metastasis. Arrowheads indicate vessel-like T2* hypointensity.

*In vivo* and* ex vivo* MRI screening of JIMT-1 showed T2*w hypointense lesions **(Figures 3A, S5A)**. This hypointense signature was predominantly located at the edge of the metastases, which we were able to show using high-resolution *ex vivo* MRI with a cryogenic coil 56 resulting in an isotropic voxel size of 30 µm **(Figure S5B-C)**. We observed an increasing number of T2*w positive lesions over time, marking a progressive worsening in the brain metastastic landscape **(Figures 3B, S5D)**.

Our *in vivo* BRIDGE pipeline revealed an association of T2* map-positive metastases with a trend toward reduced blood flow velocity in perimetastatic capillaries with diameters under 12 µm **(Figure 3C)**, which corresponds to approximately twice the size of a murine erythrocyte 57. Further, we could see a heterogeneity of this radiological signature and identified both T2* map-positive and negative metastases **(Figures S1F, S5E-F)**. Utilizing the *ex vivo* BRIDGE pipeline with additional immunofluorescence stainings for metastatic cell markers and the erythrocyte marker Ter119 58, we observed increased numbers and sizes of metastatic cell clusters (MCs) and erythrocyte clusters (ECs) in T2*w positive lesions **(Figure 3D-H)**. Importantly, we could confirm with correlative electron microscopy of our breast cancer model the presence of erythrostasis within the capillaries **(Figures 3I-J)**. This is in line with previous findings that showed a vascular remodelling of breast cancer brain metastases 59.

When comparing the proportion of T2*w/SWI hypointense breast cancer brain metastases in mice with those in human breast cancer patients measured with 3T imaging, the distribution is similar. Approximately 30% of metastases are T2*w/SWI isointense in both the mouse model and breast cancer brain metastasis patients. In humans, 18,5 % of SWI hypointense metastases appear vessel-like **(Figure 3K)**. After segmentation of the perimetastatic SWI hypointensity, the 3D visualization further confirms its vessel-like configuration **(Figure 3L)**. This supports the hypothesis that at least a portion of SWI hypointense lesions in human brain metastasis patients is caused by erythrostasis.

Together, these findings reveal a biological mechanism distinct to microbleedings that underlies the T2*w hypointensities and allows to develop novel microscopy-inspired biomarkers for radiological detection of brain metastases.

### Voxel-to-voxel correlation of MRI and intravital microscopy reveals underlying ground truth of emerging MRI signatures of glioblastoma

In glioblastoma, preceding research has led to the hypothesis that T2w hyperintensity could be an early indicator of glioma progression 26. Here, we attribute biological significance to this hypothesis.

**Figure 4:**
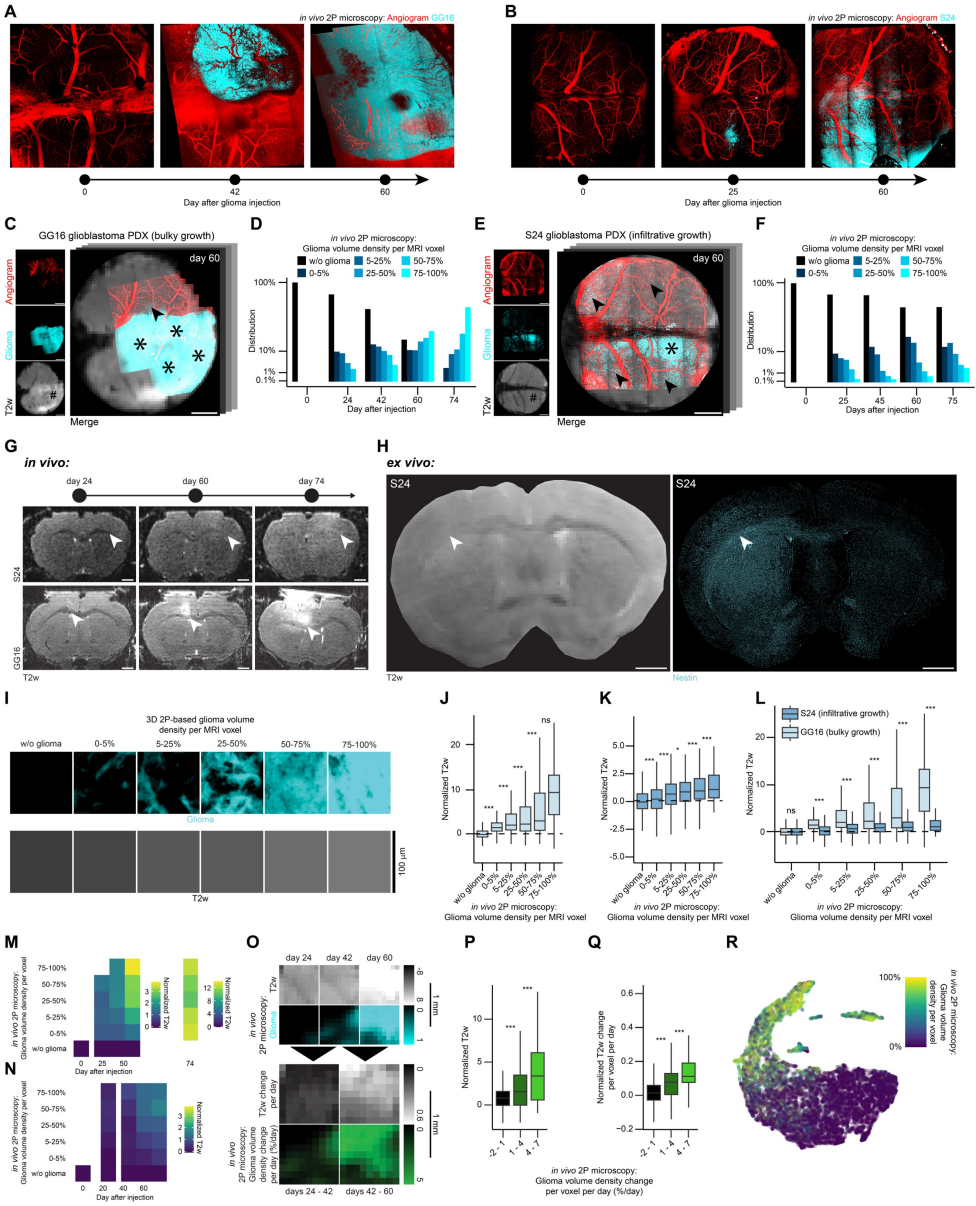
** BRIDGE reveals underlying cellular tumor burden in MRI during glioblastoma progression. A,** Timeline shows exemplary vessel architecture (red) and GG16 (cyan) at days 0, 42 and 60 after tumor implantation in the same mouse. **B,** Timeline shows exemplary vessel architecture (red) and S24 (cyan) at days 0, 25 and 60 after tumor implantation in the same mouse.** C,** *In vivo* 2P-MRI correlation of exemplary GG16 at day 60 after tumor implantation. Stars depict bulk tumor regions, arrowheads depict infiltrative regions, hashtag depicts visible tumor in T2w sequence. Scale bars: 1 mm. **D,** Barplot showing glioma volume density distribution in GG16 over time measured in 2P microscopy (n = 4 mice; day 0, n = 1 dataset, 5408 voxels; day 24, n = 3 datasets, 4636 voxels; day 42, n = 3 datasets, 7691 voxels; day 60, n = 3 datasets, 4930 voxels; day 74, n = 3 datasets, 4890 voxels). **E,** *In vivo* 2P-MRI correlation of exemplary S24 at day 60 after tumor implantation. Stars depict bulk tumor regions, arrowheads depict infiltrative regions, hashtag depicts invisible tumor in T2w sequence. Scale bars: 1 mm. **F,** Barplot showing glioma volume density distribution in S24 over time in 2P microscopy (n = 17 mice; day 0, n = 3 datasets, 11752 voxels; day 25, n = 8 datasets, 20541 voxels; day 45, n = 8 datasets, 17593 voxels; day 60, n = 11 datasets, 27689 voxels; day 75, n = 7 datasets, 18025 voxels). **G,** Development of *in vivo* T2w signal 24, 60 and 74 days after tumor injection. Arrowheads indicate fading of CC. Top: S24, bottom: GG16. Scale bars: 1 mm. **H,** *Ex vivo* co-registration of T2w image (left) and Nestin (cyan, right) in mouse brain with S24. Arrowheads indicate fading of CC (left) and high glioma density (right). Scale bars: 1 mm. **I,** Exemplary 2P-correlated MR voxels grouped based on glioma volume density with corresponding T2 voxels to visualize the correlation between glioma density and T2 signal intensity. Scale bars: 100 μm. **J,** Boxplots showing significant correlation of glioma density with normalized T2w in GG16 *in vivo*. Median with interquartile range (n = 4 mice, 13 datasets; w/o glioma, n = 14173 voxels; 0-5%, n = 2224 voxels; 5-25%, n = 2313 voxels; 25-50%, n = 2192 voxels; 50-75%, n = 2566 voxels; 75-100%, n = 4087 voxels; ANOVA with post-hoc Tukey HSD test).** K,** Boxplots showing glioma density and T2w correlation in S24* in vivo*. Median with interquartile range (n = 17 mice, 37 datasets; w/o glioma, n = 69490 voxels; 0-5%, n = 12333 voxels; 5-25%, n = 8881 voxels; 25-50%, n = 3292 voxels; 50-75%, n = 1156 voxels; 75-100%, n = 447 voxels; ANOVA with post-hoc Tukey HSD test). **L,** Boxplots showing T2 signal intensity differences of bulky growth model GG16 and infiltrative growth model S24* in vivo* depending on 2P microscopy glioma density per MRI voxel. Median with interquartile range (n values are the same as in f and g; Welch's t-tests). **M,** Heatmap of glioma density compared to normalized T2w in GG16* in vivo* (n = 4 mice, 13 datasets, 27555 voxels). **N,** Heatmap of glioma density compared to normalized T2w in S24 *in vivo* (n = 17 mice, 37 datasets, 95599 voxels). **O,** Image crops of longitudinal GG16 tumor in 2P and T2w correlation* in vivo*, highlighting voxel density changes over time. Scale bars: 1 mm. **P,** Correlation of normalized T2w signal intensity with glioma growth rate in GG16* in vivo*. Median with interquartile range (n = 3 mice, 9 datasets; -2-1%, n = 2414 voxels; 1-4%, n = 688 voxels; 4-7%, n = 197 voxels; Kruskal-Wallis test followed by Dunn-Bonferroni post-hoc test). **Q,** Change of T2w signal intensity with glioma growth rate in GG16* in vivo*. Median with interquartile range (n values same as l, Kruskal-Wallis test followed by Dunn-Bonferroni post-hoc test). **R,** UMAP dimension reduction of normalized *in vivo* T2w, T1-native, and T1-postcontrast data for GG16, labeled by glioma volume density (n = 4 mice, 13 datasets, 27555 voxels).

To investigate the dynamic changes in MRI during glioblastoma progression, two patient-derived glioblastoma models were assessed: GG16 60, characterized by early changes of the vascular architecture** (Figure 4A)** and its bulky growth **(Figures 4C-D)**, and S24 61-64 **(Figure S6A)**, showing no changes of the vascular architecture in early stages **(Figures 4B, S6B)** and a diffuse, infiltrative growth pattern **(Figure 4E-F)**. At 60 days post-injection, GG16 tumors showed visible T2w hyperintensity, whereas no tumor was detected using high-resolution MRI in the S24 model **(Figure 4C, E)**, highlighting the heterogeneous growth patterns of these models **(Figure S6F)**.

*In vivo* imaging revealed a progressive T2w signal intensity increase of the corpus callosum over time in both S24 and GG16 **(Figure 4G)**. Corresponding *ex vivo* microscopy and *ex vivo* T2w image confirmed that this T2 hyperintensity correlated with increased glioma cell density **(Figure 4H)**.

Using our voxel value extraction workflow **(Figures S1G, S6C)**, we analyzed tumor and adjacent brain parenchyma voxels **(Figure 4I)**. Both models demonstrated a significant increase in normalized T2w signal intensity corresponding to glioma volume density per MR voxel **(Figures 4J-K, S6D-E)**, even in areas of low glioma density, typically associated with infiltrative growth 63 and in regions where manual segmentation did not detect the tumor **(Figure 4E)**. In comparison of both tumor models, the bulky growth model (GG16) exhibited significantly higher normalized T2w signal intensity compared to the infiltrative model (S24) at similar glioma cell densities **(Figure 4L)**. Longitudinal analysis further showed a positive correlation of T2w signal increase with increased glioma density and time **(Figure 4M-N)**. Even though no to little vascular architecture changes occur in the infiltrative growth model S24 **(Figures 4B, S6B)**, the T2w signal intensity increases with time **(Figure 4N)**. This demonstrates that T2w signal intensity increase does not only depend on changes of the vascular architecture and associated vasogenic edema 65, 66, but can also decode infiltrative growth in the absence of vascular changes. Furthermore, longitudinal registration **(Figure 4O)** of GG16 datasets confirmed that normalized T2w signal intensity significantly correlated with glioma growth rate over time **(Figure 4P)**. Further, in GG16 increases in T2w signal intensity were significantly linked to glioma growth rate per time **(Figure 4Q)**. To summarize, there appears to be a gradient in the infiltration zone ranging from weak to strong hyperintense T2 signal. This gradient seems to correlate with glioma density and glioma dynamics, i.e. growth rate and time after glioma injection.

A UMAP dimensionality reduction 67 was employed to illustrate the correlation between MRI and 2P microscopy demonstrating how glioma density aligns with multimodal MRI data **(Figures 4R, S6G-L)**.

### *Ex vivo* expansion super-resolution microscopy-MRI correlation enhances effective image resolution of macroscopic imaging

**Figure 5:**
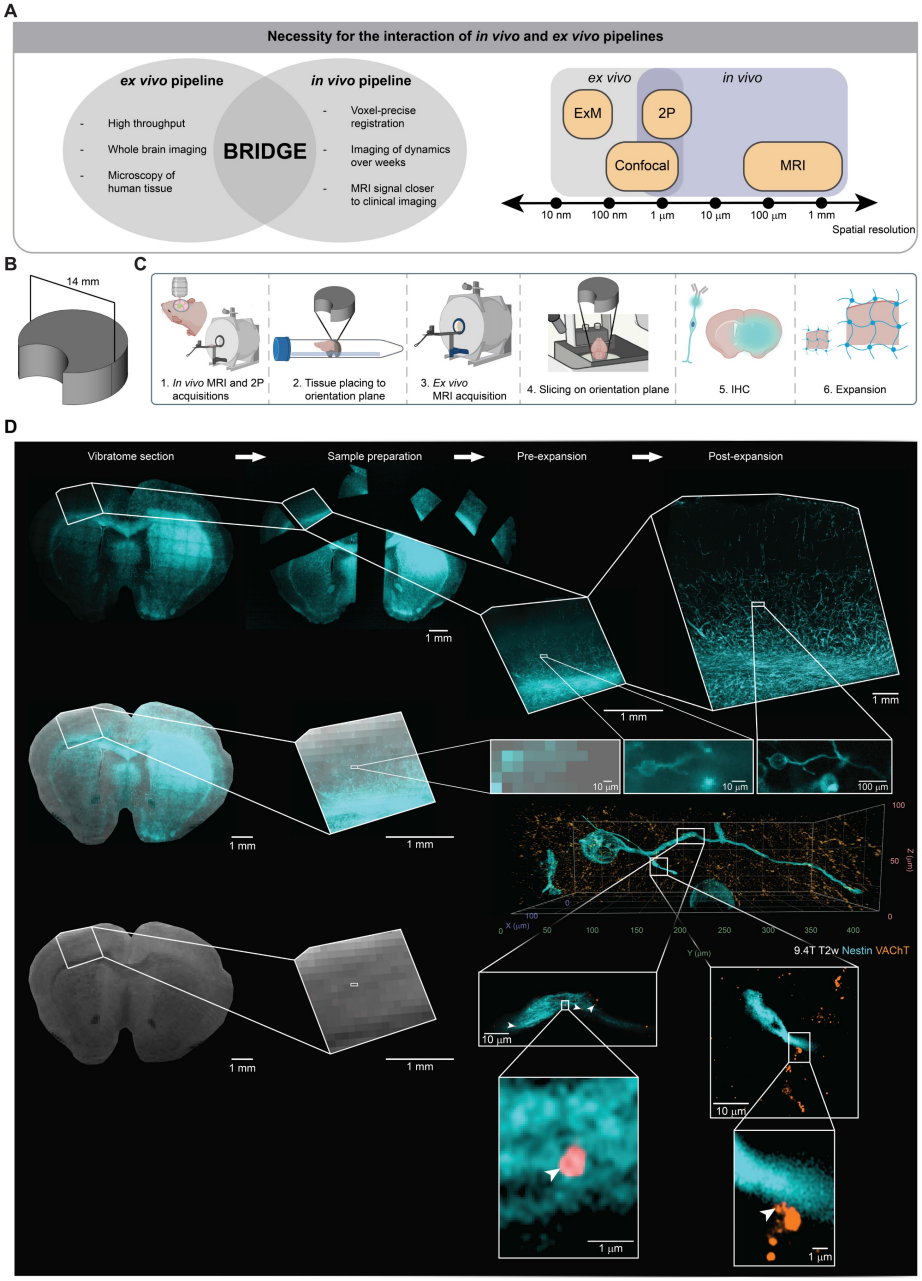
** Correlative *ex vivo* MRI and super-resolution microscopy. A,** Left: The interaction between the *in vivo* and *ex vivo* pipelines offers various advantages. Right: Spatial resolutions of imaging techniques subdivided into *in vivo* and *ex vivo* imaging. **B,** 3D model of the custom device used in the *ex vivo* correlation pipeline. **C,** Schematic of the patient-derived mouse model image acquisition pipeline across scales. **D,** Correlative *ex vivo* expansion microscopy and MRI in the patient-derived S24 glioblastoma model mouse brain. Nestin (cyan) represents glioblastoma cells and vesicular acetylcholine transporter (VAChT) is a vesicular marker of cholinergic synaptic vesicles (orange). Arrowheads depict putative neuron-to-glioma synapses (NGS).

A crucial part of this work is the integration of our *in vivo* and *ex vivo* co-registration and analysis pipelines. While the *in vivo* pipeline could track subcellular dynamics over weeks, the *ex vivo* pipeline enables high-throughput endpoint imaging of human and mouse tissue combined with whole brain imaging and super-resolution microscopy. The interface between *in vivo* and *ex vivo* imaging combines high temporal resolution with high spatial resolution enabling to bridge scales **(Figure 5A)**.

To further push the limits of image resolution with correlative MRI technologies, we integrated an *ex vivo* microscopy pipeline using expansion microscopy as a super-resolution microscopy approach 31-36. To ensure a cross-modality, standardized reference plane, we developed a custom 3D-printed reslicing device for vibratome sectioning **(Figure 5B)**. This device is specifically designed to mount brain tissue on a pedestal serving as a common orientation plane for *ex vivo* MRI as well as subsequent slicing and microscopy **(Figure 5C)**. It includes a lunar notch designed to prevent air bubble accumulation near the brain tissue, thereby reducing the risk of introducing artifacts into the MR images 68 **(Figure S7A)**. Furthermore, the stable mounting brings the *ex vivo* tissue closer to the MRI coil, enhancing the SNR 69.

Subsequent co-registration utilizes anatomical landmarks, ensuring precise alignment between MRI and microscopy. Since each brain section corresponds to the thickness of an MR voxel, aligning one MR slice with one brain slice ensures that all subsequent slices are aligned **(Figure S7B)**. After co-registration, it is possible to register the slices onto the Allen Mouse Brain Common Coordinate Framework (CCFv3) 70 **(Figure S7C)**.

Before analyzing *ex vivo* glioblastoma slices, we examined whether the Nestin antibody is specific to human Nestin. Immunohistochemistry for human-specific Nestin and intrinsic GFP in a mouse xenograft model of S24-mGFP confirmed a high degree of colocalization between the fluorescent tumor reporter signal and human Nestin expression **(Figure S7D)**. Quantitative analysis showed that 94.0% of all Nestin-positive cells exhibited intrinsic GFP signal **(Figure S7E)**. These data demonstrate that the the Nestin antibody reliably labels human glioblastoma cells within this patient-derived xenograft model in line with previously published work 71, 72.

To further push the resolution boundaries on the microscopic scale to the level of single synaptic boutons, we conducted correlative expansion microscopy with an expansion factor of 4 31. This approach enables the visualization and MRI co-registration of putative, individual neuron-to-glioma synapses (NGS) which were previously described as hallmark of multicellular tumor networks 63, 64, 71, 73 **(Figure 5D, Movie S2)**. Together, this workflow paves the way for the development of radiological predictors of malignant connectivity by enabling the quantification of NGS 63, 64, 71, 73 in MR voxels.

### Clinical translation of human brain tumor MRI signals using BRIDGE

**Figure 6:**
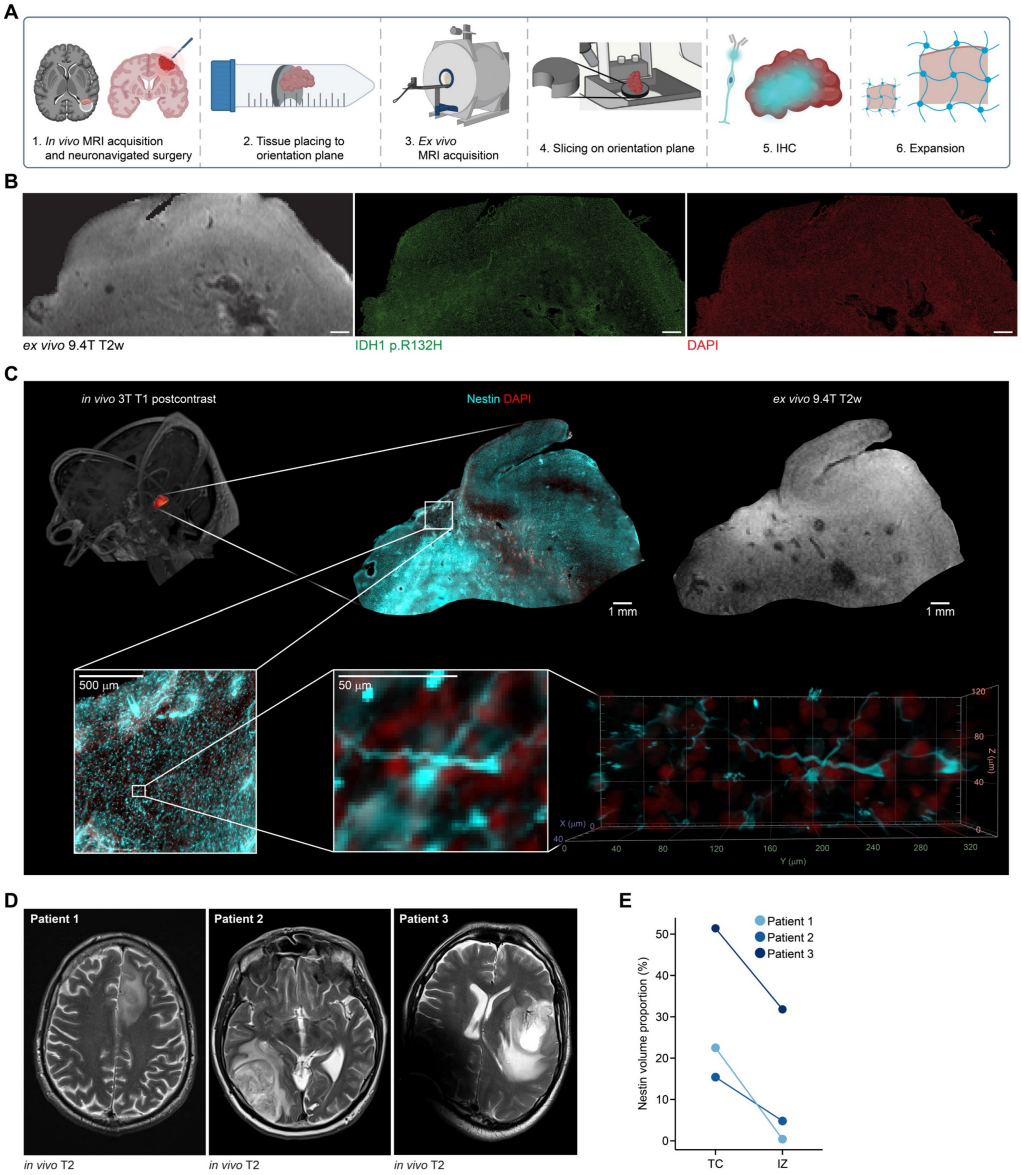
** Clinical-translational investigation of human brain tumor tissue with BRIDGE. A,** Schematic of the human acquisition pipeline to bridge scales. **B,** Co-registration of *ex vivo* 9.4T T2w sequence and tissue stained for IDH1 p.R132H (green) and DAPI (red). Scale bars: 1 mm. **C,** Correlative *in vivo* and *ex vivo* MRI and *ex vivo* super-resolution microscopy in human astrocytoma grade 4 tissue. Nestin (cyan) and DAPI (red). **D,** Clinical T2w images of three glioblastoma patients. **E,** Comparison of Nestin volume proportions of the same three patients as in (D), subdivided into TC and IZ, highlighting the heterogeneity of glioblastoma.

Lastly, we established a clinical translation pipeline to apply BRIDGE to human glioma tissue. For this purpose, we used a combination of *in vivo* 3T MRI, *ex vivo* 9.4T MRI and super-resolution microscopy to investigate human glioma tissue which was extracted via neuronavigation **(Figures 6A)**. We were able to register microscopy precisely to the T2w image using vessel landmarks **(Figure S8A)**. With this workflow, we were able to map the distribution of IDH-mutant astrocytoma cells to the T2w image **(Figure 6B)**. In our further analyses, we stained against Nestin to visualize tumor cells with their whole-cell morphology. Therefore, in the same astrocytoma patient, we showed that Nestin does not show all tumor cells (sensitivity: 58.8%), but all Nestin-positive cells appear to be tumor cells (specifity: 100%) **(Figure S8B-C)**. Leveraging super-resolution microscopy, we co-registered Nestin-positive cells with subcellular resolution to MRI voxels. This investigation enabled to visualize previously described neurite-like structures of glioma cells that are important for the invasion into brain tissue 61, 63, 71 **(Figure 6C, Movie S3)**.

Furthermore, we introduced an approach to map *ex vivo* human tissue to clinical T1 post-contrast and FLAIR MRI sequences via neuronavigation **(Figure S8D)**. We analyzed human glioblastoma tissue from tumor core (TC) and infiltration zone (IZ) and showed that the density of Nestin-positive cells in TC and IZ were highly heterogeneous in three different patients. However, the density of Nestin-positive cells in tissues of TC was higher than in IZ **(Figures 6D-E, S8E)**. Summarized, this workflow presents an approach to investigating the underlying biology of MRI signal in human glioma tissue for future investigations.

## Discussion and Conclusion

We established a framework called BRIDGE for longitudinal and multimodal voxel-accurate correlation between MRI and light microscopy, integrating *in vivo* 2P microscopy and *ex vivo* super-resolution microscopy.

Previous correlative imaging techniques have often encountered significant registration challenges, such as translation, rotation, elastic differences and resolution adjustments. Traditional methods relying on brain slice contours as landmarks 74 or positioning the brain within pathology slice blocks 75 have shown inaccuracies. More advanced techniques, like 3D-3D registration with CLARITY 76 lack longitudinal registration and therefore the ability to track changes. *In vivo* correlative imaging has attempted to use vascular landmarks to align MR angiograms with microscopic imaging 18, offering higher temporal resolution, but often sacrificing spatial precision, especially in terms of axial resolution when widefield fluorescence imaging was used. BRIDGE addresses these limitations by improving spatial registration accuracy through an iterative registration pipeline. Our workflow provides consistent and reliable landmarks for multimodal registration using uniquely identifiable vessel branches visible in 3D on both angiograms and MRI. We evaluated MR sequences concerning the visibility of possible landmarks and found T2w and T2*w sequences to be the most suitable sequences.

Additionally, the integration of BRIDGE into *ex vivo* settings is implemented with a custom 3D-printed device that minimizes reslicing steps, enabling faster and more accurate registration in the x, y and z axes. However, *in vivo* 2P imaging remains limited by its 600-800 µm penetration depth 77, restricting whole-brain coverage. The *ex vivo* pipeline offers whole brain imaging and high resolution but cannot track longitudinal changes. Therefore, bridging *in vivo* and *ex vivo* pipelines become essential for combining dynamic monitoring with high-resolution and whole brain analysis.

BRIDGE enables the development of AI-driven MRI predictions using microscopic information as ground truth. Above all, it allows to use any microscopic contrast for training AI algorithms. Previously developed segmentation methodologies, predominantly relying on manual or semantic annotations, showed the potential of using neural networks for medical image segmentation 78, 79. Our approach substantially augments image quality and reduces the reliance on human input, improving overall robustness and reproducibility. Without co-registered microscopy, segmentation is limited to the voxel level, as manual segmentation cannot account for hidden details within each voxel. Integrating microscopy allows deep learning to enhance segmentation beyond MRI resolution. We were able to refine and improve the resolution of MR images revealing near microscopic information of the vascular architecture in MRI. Our AI-driven analysis holds promise for training models using BRIDGE's voxel-accurate co-registration. As these technologies evolve, BRIDGE could be translated to human tissue studies, opening new clinical opportunities for comprehensive *in vivo* imaging and diagnostics. We showed a proof-of-concept in this manuscript how to co-register human glioma tissue on MRI, paving the way for BRIDGE's path to clinical translation.

Further, BRIDGE enables the extraction of correlative voxel intensity values, providing quantitative insights into structural changes over time. We assessed brain vessel visibility across different MRI sequences and correlated these findings with high-resolution 2P imaging. The use of a cryogenic coil enabled the detection of microvascular structures down to 20 µm, improving vessel segmentation and quantitative analyses. The distinct correlation patterns observed between TOF, T2w and T2*w sequences with vessel diameter and blood flow velocity provide valuable insights into the strengths and limitations of each modality for vascular imaging in living brain tissue.

In the context of brain metastases, our technology platform allowed the characterization of the role of erythrostasis and microvascular changes in T2*w hypointense lesions. The association between T2* map positivity and reduced perimetastatic blood flow suggests that vascular dysfunction may contribute to metastatic progression. Furthermore, *ex vivo* analyses confirmed increased metastatic and erythrocyte cluster formation in T2*w hypointense lesions, reinforcing the link between vascular pathology and metastasis 59. These insights emphasize the potential of MRI-based vascular biomarkers in monitoring tumor-associated vascular changes and guiding future therapeutic strategies. Importantly, we could also see similar T2*w hypointense lesions in human patients which will need further investigation in the future.

BRIDGE is able to track dynamic MR signal intensity changes of single voxels during glioblastoma progression. The distinct MRI characteristics of GG16 and S24 reflected their different growth patterns, with GG16 showing higher normalized T2w signal intensity even at similar glioma densities in 2P. Longitudinal analysis revealed that T2w variations depend on glioma density and time since tumor injection as well as tumor growth rate, subdividing hyper- and isointense glioma infiltration zone. These findings support voxel-based MR analysis as a valuable tool for monitoring glioblastoma progression and improving imaging-based tumor assessment.

In summary, BRIDGE provides a longitudinal, voxel-accurate correlation of MRI with both *in vivo* and *ex vivo* microscopy. By overcoming longstanding challenges in multimodal registration and expanding the potential of high-resolution imaging, BRIDGE stands as a powerful tool for translational research and will be able to affect clinical diagnostics by improving MR imaging through ground truth based deep learning.

## Methods

### Study participant details

Human tissues were obtained after approval of the local regulatory authorities (ethical codes S-458/2021, S-672/2023, 23-1233-S1, 23-1234-S1, S-005/2003, 23-1175-S1 and PV4904). Human patient samples were pseudonymized manually.

### Cultivation of patient-derived primary tumor cell lines and Illumina 850k methylation array characterization

Patient-derived glioblastoma cell lines were cultured from resected tumors as described in previous studies 61, 63, 71. These cells were grown in DMEM/F-12 under serum-free, non-adherent, 'stem-like' conditions, supplemented with B27 (12587-010, Gibco), insulin, heparin, epidermal growth factor, and fibroblast growth factor.

Brain-tropic JIMT-1 (human breast cancer, ER-, PR-, HER2 amplification, trastuzumab-resistant, p53-/-, a kind gift from Patricia Steeg), were cultured in DMEM supplemented with 10% fetal bovine serum (FBS) and 1% penicillin/streptomycin (pen/strep) 80-82.

The molecular classification of the tumor xenograft models used in this study is available in Table S1. DNA methylation analysis of over 850,000 CpG sites in all cell lines was performed using the Illumina Infinium Methylation EPIC kit at the Genomics and Proteomics Core Facility of the German Cancer Research Center in Heidelberg, Germany 83. Glioblastoma cell lines maintained under stem-like conditions and JIMT-1 were transduced with lentiviral vectors expressing membrane-bound GFP using the pLego-T2-mGFP construct, as described before 63, 84. The brain-tropic metastasis cell lines were stably transduced with lentiviral vectors for imaging purposes, with cytosolic expression of GFP or tdTomato achieved through transduction with plKO.1-puro-CMV-TurboGFP (SHC003, Sigma-Aldrich, USA) or cytoplasmic tdTomato (LeGo-T2, plasmid #27342, Addgene, USA). Throughout the study, all cell lines were tested for mycoplasma contamination by PCR every three months.

Regular FACS sorting of transduced cells was conducted using the FACSAria Fusion 2 (Bernhard Shoor) or FACSAria Fusion (Richard Sweet) systems. GFP-positive cells were sorted using the BL530/30 filter **(Figure S6A)**.

### Animal procedures

All animal procedures were conducted in accordance with the European Directive on animal experimentation (2010/63/EU), the Society for Laboratory animal Science (GV-SOLAS) guidelines and institutional laboratory animal research guidelines. The study was approved by the Regierungspräsidium Karlsruhe, Germany under the license numbers G50-19 and G220-16.

Male NMRI nude mice (8-12 weeks old) were used for studies involving human patient-derived primary glioblastoma cells. The S24 cell line exhibits an infiltrative growth pattern characteristic of the malignancy in human disease, while GG16 represents a tumor that is clearly visible on MRI.

Female Athymic nude mice and NSG mice (8-12 weeks old) were used for all studies involving brain metastasis models. JIMT-1 cells were cultured as adherent cultures in DMEM-high glucose media supplemented with FBS and Penicillin/Streptomycin, as previously described. All cell lines were regularly sorted for fluorescence before animal procedures.

Animals were housed at the animal facilities of the German Cancer Research Center in Heidelberg.

Surgical procedures were performed according to established protocols 61-64, 71. Custom-made teflon rings instead of conventional titanium rings 15 and bespoke alignment of cross-modality *in vivo* and *ex vivo* imaging were established to ensure MRI compatibility and initial image registration **(Figure S1A)**. For MR imaging, custom-made rings crafted from Teflon were used in place of the traditional custom-made titanium ring typically employed for painless head fixation during imaging. Previous studies have reported hyperintense areas under the window caused by local edema or scar tissue shortly after cranial window surgery 16. Our observations indicate that these artifacts regress 2-3 weeks post-surgery, making this the optimal time for correlative imaging **(Figure S1B)**.

In the xenograft glioma model, 50,000 to 100,000 tumor cells were stereotactically injected into the mouse cortex at a depth of approximately 500 µm, 1-3 weeks after cranial window surgery. 20 mice underwent injection with S24 glioma cells, four mice got injected with GG16 glioma cells. Alternatively, glioma cells were injected stereotactically into the striatum without prior cranial window surgery.

For brain metastasis models, tumor cells trained in brain tropism were suspended in phosphate-buffered saline and injected into the left cardiac ventricle, following established protocols 59, 82, 85. Four mice underwent intracardial injection with JIMT-1 breast cancer metastasis cells.

### Intravital microscopy

Mice that had previously undergone cranial window surgery and tumor injection were observed intravitally for up to 150 days in the glioma model and for 28 days in the brain metastases model following tumor implantation. Imaging was performed using a Zeiss 7MP microscope and a Zeiss LSM 980 with Airyscan2, both equipped with a pulsed Ti:Sapphire laser (Chameleon Discovery NX; Coherent). Fluorescent markers, including GFP, tdTomato, FITC, and TRITC dextran, were imaged using 850 nm and 960 nm wavelengths, respectively. The microscopes were configured with bandpass filter sets of 500 - 550 nm and 575 - 610 nm. A 10x, 0.45 NA and 20x, 1.0 NA, apochromatic, both with 1.7 mm working distance, water immersion objectives (Zeiss) were used for imaging on both setups. Fluorescence emission was detected using low-noise, high-sensitivity photomultiplier tubes.

Mice were anesthetized with isoflurane gas diluted in 100% O^2^. Anesthesia was induced with 3-5% isoflurane and reduced to 0.5-1.5% for maintenance during imaging, monitored by the breathing rate. Eye cream was applied to protect the eyes after anesthesia induction. Throughout the imaging process, the body temperature of the mice was maintained at 37 °C using a temperature sensor and heating plate. Anesthesia depth was regularly assessed by monitoring the mice's posture and breathing rate.

For the angiogram, TRITC-dextran (500,000 g/mol) was dissolved in a 0.9% NaCl solution at a concentration of 10 mg/ml. Before imaging, 100 µl of the TRITC or FITC solution was injected into the lateral tail vein.

Tile scans of the entire brain surface were acquired for the correlation pipeline using the 10x objective, 0.45 NA, apochromatic, water immersion, with pixel size of 1.1838 µm.

For Airyscan imaging, TRITC was used to visualize blood vessels, while the region of interest was selected based on the GFP channel, specifically highlighting the JIMT-1 mGFP-expressing cells. Imaging was conducted using a 20x, 1.0 NA, apochromatic, water immersion objective (Zeiss) with a 1.7 mm working distance. The pixel size utilized was 59.54 nm, providing high-resolution imaging.

For blood flow velocity measurements, intravital imaging was performed using an intravenous angiogram with TRITC as previously described. Imaging was conducted with a 20x, 1.0 NA, apochromatic, water immersion objective (Zeiss) with a 1.7 mm working distance. A region of interest containing the vessels of interest was selected. A line scan was placed parallel to the vessel, consisting of 128 pixels with a pixel size of 1.1838 µm, resulting in a total line length of 10.6066 µm. The line was scanned repetitively for 1 second with a frame interval of 0.15 ms.

The data was processed in Zen software to generate an xy-image. Subsequent analysis was performed using a custom-written macro in Fiji, where 10 lines per line scan were drawn manually corresponding to the angle of the recorded blood flow. The mean blood flow velocity and the standard deviation were calculated.

### Intravital MRI

MRI scans were conducted using a 9.4T horizontal bore small animal MRI scanner (BioSpec 94/20 USR, Bruker BioSpin GmbH, Ettlingen, Germany) which was outfitted with a gradient strength of 675 mT/m. For S24 mice, acquisitions were conducted using an 8.4 cm body coil for transmission and a receive-only 4-channel surface array coil. Scans of mice with GG16 and JIMT-1 were acquired using a cryogenic RF coil. To induce anesthesia, 4% isoflurane in 100% oxygen was used. During scanning, 1-1.5% isoflurane in 100% oxygen was administered via a nose cone. The respiratory rate was continuously monitored, and the animals were placed on a Bruker standard MRI bed with an integrated water circulation heating system to maintain body temperature. The test animals were administered a maximum of 0.1 ml of contrast agent (0.5 mmol/ml Gadolinium diluted 1:5 in PBS) intravenously. The sequences are listed in Table S2.

### Human MRI

MRI examinations were performed on two 3T clinical scanners (Siemens Healthineers, Magnetom Skyra and Magnetom Prisma). The Magnetom Skyra eco was equipped with gradients of 45 mT/m and a slew rate of 200 T/m/s, whereas the Magnetom Prisma provided a maximum gradient strength of 80 mT/m at 200 T/m/s and included an XR 80/200 gradient coil. The imaging protocol for all participants comprised 3D T1-weighted MmPRAGE acquisitions obtained before and after administration of a gadolinium-based contrast agent (Clariscan™, 0.5 mmol/ml), along with 3D FLAIR and SWI sequences.

### Transcardial perfusion

Brain perfusion was performed as previously described 61, 63. Tumor-bearing mice were anesthetized using a combination of ketamine and xylazine. After confirming the absence of interphalangeal reflexes, the animals were transcardially perfused with 4% paraformaldehyde (PFA) and PBS. Following brain extraction, the tissue was fixed in 4% PFA overnight and then stored in PBS.

### 3D print device

We created a self-designed, easy-to-print and -use reference plane for reslicing by using the software FreeCAD and UltiMaker Cura and the 3D printer UltiMaker 2. The device consists of PLA filament. The main purpose of this device is to find the same slicing angle in MRI and microscopy. Besides that, the device solves the fixation of the brain to reduce MRI artifacts and gets the brain as close to the coil as possible. To use this device, the brain tissue is first cut between the midbrain and cerebellum to create a flat surface. The cut brain gets sticked on the self-designed device with superglue. Subsequently, it is pushed into a 15 ml centrifuge tube where it fits perfectly. The tube is filled with PBS and sealed so that as little air as possible remains in the tube. However, as a little air can remain in the tube, the 3D-printed device also serves as a bubble trap during MRI scans **(Figure S7A)**. To get the brain out of the tube, a simple wire was used to pull on the small opening in the device. Following, the brain is sliced on the same device to find the same reslicing angle in microscopy.

### *Ex vivo* 9.4T MRI acquisition

*Ex vivo* 9.4T MRI scans were conducted using the same scanner as described for *in vivo* scans. Scans were acquired using a cryogenic RF coil. To place the tissue as close to the coil as possible, the tissue gets sticked on the 3D print device. The sequences are listed in Table S3.

### Vibratome slicing of brain tissue

After *ex vivo* MRI acquisition, the brain tissue, which was glued onto the 3D-printed device, was removed from the 15 ml falcon tube. The 3D-printed device was then directly attached to the vibratome holding device. Brain slices, 80-100 µm thick depending on MRI voxel size, were cut using a vibratome (Leica VT1000S) to align with the voxel size of the MRI.

### Immunohistochemistry

Brain slices were either permeabilized with 5% FBS and 1% Triton X-100 (TX100) for 2 hours at room temperature or underwent antigen retrieval using 20 mM sodium citrate, incubated at 60°C for 1 hour. After antigen retrieval, the slices were washed with 10% FBS.

Primary antibodies were incubated in 1% FBS and 0.2% TX100 (anti-GFP chicken, Abcam ab13970, 1:1000; anti-RFP guinea pig, SySy REF#390004, 1:200; anti-Ter119 rat, Thermo Fisher Invitrogen, REF#14-5921-85, 1:500, anti-IDHR132H mouse, Dianova, REF#DIA-H09, 1:2) overnight on a shaking device at 4°C. Slices were then washed twice in 2% FBS, followed by incubation with the secondary antibody at a 1:500 dilution in 1% FBS and 0.2% TX100 for at least 3 hours at room temperature. After secondary antibody incubation, slices were washed three times with 1% FBS and then three times with PBS. Finally, slices were washed with 1:10000 DAPI in PBS before widefield microscopy. Slices were mounted on microscope slides with SlowFade gold.

### Human immunohistochemistry

Brain slices either underwent antigen retrieval by heating 20 mM sodium citrate buffer in a microwave until boiling, immediately transferring the hot buffer to the wells containing the tissue and then incubated at 60°C for 30 minutes or permeabilized with 5% FBS and 1% Triton X-100 (TX100) for 2 hours at room temperature. Following antigen retrieval, slices were blocked in 5% FBS in PBS for 2 hours at room temperature.

Primary antibody incubation was performed overnight at 4°C on a shaker in a solution containing 1% FBS and 0.2% Triton X-100 in PBS (anti-Nestin, mouse, 1:300, Abcam, ab22035, anti-CD31, goat, R&D systems, AF3628, 1:100). After incubation, slices were washed three times for 10-15 minutes each with 2% FBS in PBS.

Secondary antibodies (Alexa Fluor 488 donkey anti-mouse for Nestin; Alexa Fluor 647 donkey anti-rat for CD31) were diluted 1:500 in 1% FBS in PBS (without Triton X-100) and incubated either overnight at 4°C or for 3 hours at room temperature on a shaker. After secondary antibody incubation, slices were washed three times for 10-15 minutes each in 1% FBS in PBS, followed by a final 10-15 minute wash with PBS alone.

For expansion microscopy, only Nestin-stained slices were used, and the Alexa Fluor 488-conjugated secondary antibody was replaced by Alexa Fluor 568. Finally, all slices were mounted with SlowFade Gold containing DAPI and imaged using widefield fluorescence microscopy.

### Widefield microscopy of *ex vivo* slices

Widefield microscopes (Leica DM6000, Leica Mica, Zeiss AxioScanZ1) were used to acquire multichannel tile scans of stained *ex vivo* slices. For quantitative analyses, the brain slices were imaged at the AxioScanZ1 microscope with a pixel size of 325 nm using a 20x (NA 0.8) objective.

### Expansion microscopy

Brain slices were prepared and stained as described in the immunohistochemistry section, with the exception of using Atto 647N instead of Alexa 647, and staining endogenous mGFP with anti-GFP as described previously. Both primary and secondary antibody incubation times were extended to 24 hours. Following staining, slices were screened using a widefield fluorescence microscope (Leica DM6000), and small regions of interest, approximately 1 cm x 1 cm in size, were cut out using a scalpel.

For sample anchoring, the trimmed tissues were incubated in 0.1 mg/ml Acryloyl-X SE (AcX) solution diluted in 1x PBS overnight at room temperature without shaking. Right before the gelation procedure, a gelation chamber was prepared by placing two 1.5 coverslips, cut with a diamond knife, on top of each other at the edges of a parafilm-coated objective slide. The samples were then placed onto a Poly-L-Lysin-coated objective slide.

To prepare the gelation solution, 470 μl of monomer solution (composed of 0.08% [v/v] sodium acrylate [33% wt stock], 2.5% [v/v] acrylamide [50% wt stock], 0.02% [v/v] cross-linker [1% wt stock], 1.9M NaCl [5M stock], 1 ml of 10x PBS, and 18.8% [v/v] water) was combined with 10 μl each of 0.5 wt% 4-HT, 10 wt% TEMED, and 10 wt% APS. All stock solutions were prepared with MilliQ water. Then, 60 μl of the freshly prepared gelation solution was applied between the spacers, and the Poly-L-Lysin slide with the tissue was placed on top of the gelation solution. Radical polymerization was initiated by incubating the gelation chamber at 37°C for 2 hours.

Post-polymerization, the gel was trimmed into a trapezoid shape to distinguish the orientation of the sample and transferred into a 12-well plate containing 1 ml of digestion buffer (for 50 ml: 250 μl Triton-X100, 100 μl EDTA, 2.5 ml Tris-Cl, 2.338 g NaCl, filled up with MilliQ water - with 1:100 Proteinase K [800U/ml] freshly added before the experiment). The samples were incubated overnight at room temperature on a shaker.

The next day, the gel was washed twice with 1x PBS for 30 minutes each. Subsequently, the gel was transferred into a 6-well plate with MilliQ water. The water was exchanged every 10 minutes for three cycles, followed by an additional 30-minute wash. The samples were then trimmed and mounted on Poly-L-Lysin-coated glass-bottom dishes (MatTek) for imaging, and were covered with MilliQ water.

For Airyscan microscopy, the expanded sample was imaged using an LSM900 Airyscan NIR microscope (Zeiss) equipped with a 40x, 1.2 NA water immersion objective. Images were acquired with a pixel size of 59.54 nm. Airyscan post-processing was performed using Zen Blue software.

### Electron microscopy

The procedure for sample preparation was carried out in accordance with previously established methods and experimental data from Venkataramani et al., 2024 were re-analyzed in the course of this study 82.

### Content-aware image restoration

For content-aware restoration, training data was collected following established methodologies 63 and trained using CSBDeep 25. The prediction outputs were utilized as prediction maps in Ilastik 39. Subsequently, the raw data was segmented using Ilastik Autocontext. Registered probability maps were then binarized to values 0 and 1 using a threshold of 10000 in Fiji and converted to 32-bit. 2P stacks were grouped in z axis by average to match MRI voxel depth so that one single 2P voxel depicts the glioma volume density from 0 to 1.

### *In vivo* registration workflow

The registration workflow consists of the following steps: (1) resampling of 2P z-slices by grouping them to match the voxel depth of the MRI, (2) orientation alignment of both 2P and MR images using the cranial window glass as a common reslicing plane, (3) spatial resolution resampling of MRI in xy to match 2P pixel size, and (4) elastic, landmark-based registration, enabling precise voxel-to-voxel correlation.

To be more specific, both MRI and 2P data underwent manual reslicing in Fiji. The Reslice function was used to align the data parallel to the cranial window, ensuring that both MRI and 2P images shared the same 3D orientation which is of particular importance for our workflow. The MRI data were then upscaled to match the resolution of the 2P data in the x and y axes. Subsequently, elastic registration was performed using the thin-plate mode of the “Landmark registration” module in 3D Slicer. Affine and rigid transformations were implemented following standard methodologies 86 while elastic registration was performed using free-form deformations 87. Landmark-based registration was guided by the principles of thin-plate splines 88. The MR image was used as fixed volume, the 2P image as moving volume. T2w images were used for registrations of the glioma cohorts, T2* maps were used for registrations of the metastases cohorts. Manual landmark registration utilizing anatomical landmarks of blood vessels identifiable in both MRI and 2P imaging enables a precise registration of both vessel and tumor channels to match MRI. For local refinement, the Simple ITK option was selected. Final transformation was conducted using the 3D Slicer module “Resample Scalar/Vector/DWI Volume” by providing the transformation file resulting from the previous module, the MRI image as fixed volume and the 2P image as moving volume.

### MRI image processing

To ensure consistency of weighted imaging sequences across datasets for *in vivo* 2P-MRI correlation, MR normalization was conducted using a normal appearing gray matter z-normalization as only gray matter was analyzed 89 **(Figure S1D)**. Therefore, custom masks of every registered dataset were created based on 2P signal excluding tumor signal, vessels, lesion artifact due to injection and other artifacts and dividing the mask into two categories: normal appearing gray matter (category 'w/o glioma' in Figure 4) and glioma (other categories in Figure 4). These masks were also used to determine voxels for quantification. Mapping sequences were fitted using the Fiji plugin FijiRelax 90. Different sequences were registered using a custom-code registration Fiji macro relying on rotation and translation.

### Voxel intensity extraction

To enable a voxel-by-voxel correlative quantification, x and y axes of 2P were downscaled by bilinear interpolation to match the MRI resolution **(Figure S6C)**. The resulting downscaled 2P image consists of values from 0 to 1 representing the glioma density within this voxel. Using a self written Fiji macro, voxel intensities of the normalized MR and 2P images were extracted inside the mask described above.

### Longitudinal registration and quantification of tumor growth

After registration of each dataset on its own, the datasets of each mouse were registered to the first time point using a self-written Fiji macro relying on rotation and translation. This step enabled defining MRI and 2P value changes per day. Masks for quantification of glioma growth were created by overlapping the masks of every timepoint per mouse.

### RMSE quantification for sequence comparison

Root mean square error (RMSE) for sequence comparison was detected by defining 5 corresponding landmarks in MRI and 2P microscopy after registration. RMSE was calculated as follows:



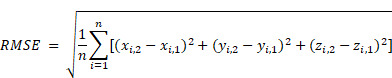



### Inter-rater reliability

Two raters familiar with the workflow registered the same six 2P microscopy datasets to already resliced T2w images. To determine interrater reliability, a coordinate system consisting of 50 columns and 50 rows per slice were applied to the grouped, but not resliced 2P images. Using this coordinate system which gets transformed during reslicing and registration, we were able to define the average RMSE per dataset between the same randomly suggested coordinates of both rater's registrations with a self-written Fiji macro.

### *Ex vivo* registration workflow

Similar to the *in vivo* registration pipeline, MRI scans were resliced in Fiji using the 3D-printed device as a reslicing plane. The x and y axes of the MRI images were upscaled to match the resolution of the microscopy images. There was no need to reslice the microscopy images, as they were already captured with the correct reslice angle due to the use of the 3D-printed device. Subsequently, the corresponding MRI slices were extracted that matched the widefield image slice stack. Since the voxel depth of the MRI and the slicing depth of the brain sections were identical, the subsequent slices were automatically aligned.

For the next step of elastic registration, the MRI images were used as fixed volumes, and the microscopy images as moving volumes. The ThinPlate mode of the "Landmark Registration" module in 3D Slicer was utilized for this process. For local refinement, the Simple ITK option was selected. The DAPI channel of the widefield images was used for registration to identify anatomical landmarks such as the corpus callosum or the anterior commissure. In most cases, a few key landmarks at prominent points were sufficient to achieve perfect registration between the modalities.

The final transformation was conducted using the 3D Slicer module "Resample Scalar/Vector/DWI Volume" by providing the transformation file generated from the previous module, with the MRI image as the fixed volume and the microscopy image as the moving volume. Landmark registration was then applied to the other channels of the widefield images. All registered widefield images were subsequently processed using Ilastik pixel classification, generating probability maps as the output.

### Semi-automatic quantification of brain metastases

*Ex vivo* JIMT-1 tdtomato brain slices stained with anti-RFP, anti-Ter119 and DAPI were registered onto T2*w MRI and probability maps were trained in Ilastik. Manual ROIs were drawn and labeled according to specific metastases. Images were thresholded and the function Analyze Particles was applied to the ROIs to measure metastatic cell clusters and erythrocyte clusters in custom written Fiji macros. The resulting measurement tables were subsequently merged and analyzed in R.

### *In vivo*-*ex vivo* registration workflow

*In vivo* and *ex vivo* MRI were elastically registered in 3D Slicer using the *ex vivo* MRI as the fixed volume. Thereby, *in vivo* MRI is automatically registered onto light microscopy data using* ex vivo* MRI for bridging between light microscopy and* in vivo* MRI.

### *Ex vivo* atlas registration

To register brain sections onto the Allen Mouse Brain Common Coordinate Framework (CCFv3) 70, we used the QUINT workflow 91 **(Figure S7C)**.

### Deep learning workflow in BRIDGE

The datasets, registered using the BRIDGE pipeline, were resampled to achieve uniform voxel spacing in the XY plane with a voxel size of 25 µm, while maintaining a voxel size of 100 µm in the Z direction. We established a minimum vessel diameter filter at 30 µm for 2P data as the approximate visibility threshold for vessels in the T2w sequence **(Figure 2F)**. This protected the neuronal network from a possible confusion caused by many small vessels **(Figure 2H)**.

Small vessels less than 30 μm in diameter were excluded to avoid confusion of the network. Therefore, a custom Fiji macro was developed to filter out structures with diameters below 30 µm. The macro systematically analyzed the local neighborhood of each voxel to estimate the vessel width. By retaining only vessels meeting the size threshold, the filtering process enhanced the relevance of the data for subsequent analysis and model training. Single slice crops were manually selected from regions with high registration accuracy and clear vessel visibility, ensuring the inclusion of areas with optimal image quality and well-defined vasculature. Vessel signal was binarized to create vessel segmentations. To increase data diversity and improve model generalization, random flips and rotations were applied as data augmentation. These transformations generated additional variations of the original data, reducing overfitting risks and enhancing the robustness of the trained model. In total, we generated 74 training datasets from 22 correlative datasets and 11 mice. We used the 2D training workflow in nnU-Net 24 to train the MR images with the preprocessed ground truth. To assess the training, we performed a 5-fold cross-validation 51 **(Figure S4B)**. We used the Dilated Dice coefficient 52 since the structures we evaluated were small and thin. In the calculation, the prediction is compared to the registered microscopy ground truth, so the Dice coefficient would also include the registration error. To compensate for the registration error, we used the Dilated Dice coefficient instead of the Dice coefficient. For dilation, 50 µm were added on both sides of the vessels. The Dilated Dice coefficient was calculated as follows:



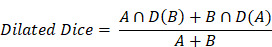



A is the microscopy ground truth, B is the prediction, D(A) is the dilation of the microscopy ground truth and D(B) is the dilation of the prediction.

Further, the mean absolute surface distance (MASD) 53 was calculated as follows:



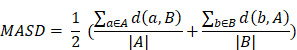



A is the microscopy ground truth, B is the prediction. a is a voxel in the microscopy ground truth, b is a voxel in the prediction. d(a,B) is the distance of a voxel in the ground truth to the closest voxel of the prediction. d(b,A) is the distance of a voxel in the prediction to the closest voxel of the ground truth. |A| is the number of ground truth voxels. |B| is the number of prediction voxels.

Additionally, all vessels in the MR image and the prediction were classified into three categories: "strongly visible," "barely visible," and "not visible." Their diameters were also compared to the ground truth. Vessel diameters in MRI were determined by thresholding the MR image.

### Quantitative evaluation of vessel visibility changes

To evaluate vessel changes in the T2w sequence, proportional distributions at timepoints 2 and 3 were compared against timepoint 1 as reference. An a priori equivalence margin of ±5% was defined. To confirm comparable vascular contrast enhancement, we quantified signal intensity changes in the sigmoid sinus on pre- and post-contrast T1-weighted images. Percent signal enhancement was calculated as follows to evaluate homogenous distribution of contrast agent: PSE = (SI_post_ - SI_pre_)/SI_pre_ × 100%. To assess whether the vessel diameter distribution remained stable across datasets, an equivalence approach was applied to the proportional data. For each diameter category, proportions at later time points were compared against the reference dataset. An a priori equivalence margin of ±5% was defined, representing the maximum biologically irrelevant deviation. A diameter category was considered stable if the maximum absolute deviation from the reference proportion across all time points remained within this equivalence margin.

### Statistical analyses

Plots were generated with the ggplot2 package and statistically tested with ggpubr from R with RStudio. Boxplots indicate minimum and maximum, median, and the first and third quartiles. For data visualization in Plots, further packages were used: gridExtra, umap, viridis, fields, RColorBrewer, plotly and cowplot.

* = P < 0.05; ** = P < 0.01, *** = P < 0.001.

### 3D visualization

3D image stacks were loaded into arivis 4D. Pixel sizes and contrast thresholds were adjusted. For visualizing MRI in 3D, skull-stripping was performed using a custom trained Unet-model. High resolution images were then taken in the ZEISS arivis software.

### Quantitative evaluation of colocalization

To evaluate colocalization of Nestin/GFP and Nestin/IDH1 p.R132H, DAPI signals of Nestin, GFP or IDH1 p.R132H positive cells were segmented and extracted. Subsequently, these positive nuclei were counted.

## Supplementary Material

Supplementary figures, table and movie legends.

Supplementary tables.

Supplementary movie 1.

Supplementary movie 2.

Supplementary movie 3.

## Data Availability

Source data are provided with this paper **(Table S8)**. Raw data are available upon reasonable request.

## Abbreviations

MRI: magnetic resonance imaging
2P: two-photon microscopy
SNR: signal-to-noise-ratio
FLAIR: fluid-attenuated inversion recovery
TOF: time-of-flight imaging
T1w: T1-weighted sequence
T2w: T2-weighted sequence
T2*w: T2*-weighted sequence
MASD: mean absolute surface distance
NGS: neuron-to-glioma synapse
TC: tumor core
IZ: infiltration zone
AI: artificial intelligence
ROI: region of interest
ExM: expansion microscopy
IHC: immunohistochemistry
VAChT: vesicular acetylcholine transporter
