# Supplementary material for: Voxel-accurate MRI-microscopy Correlation Enables AI-powered Prediction of Brain Disease States
Source: Theranostics. 2026 Mar 17;16(10):5440–62. doi: 10.7150/thno.125235 (PMC13080658; doi:10.7150/thno.125235)
Supplement: Supplementary file 1 — Supplementary figures, table and movie legends. [file thnov16p5440s1.pdf]

Supplementary material

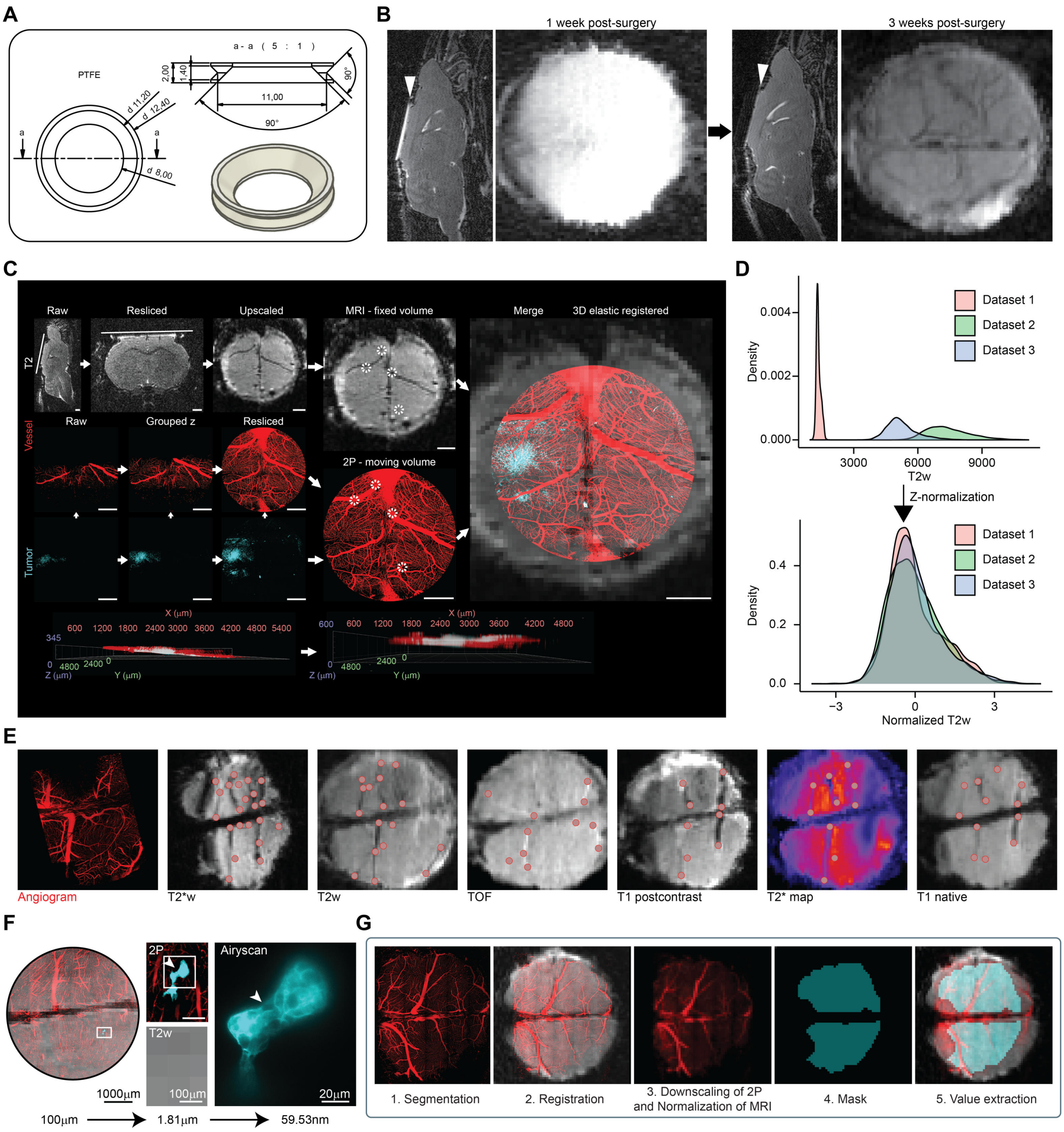

Figure S1 – *In vivo* BRIDGE workflow

A, Model of custom head fixation ring for 2P made out of Teflon (PTFE). Values in mm. **B,** *In vivo* MRI (T2w) of the same mouse 1 week and 3 weeks post-surgery. Arrowheads indicate cranial window glass. Scale bars: 1 mm. **C,** Multistep workflow including reslicing MRI and 2P images, rescaling, and elastic 3D landmark registration in 3D Slicer using blood vessel landmarks - visualized in 2D. Scale bars: 1 mm. **D,** Density plots of MR voxel value distribution

of different mice before (top) and after (bottom) z-normalization on healthy brain tissue MR voxel values (n = 3 mice, 3 datasets, 15447 voxels). **E,** Comparison of vessel branching points as possible landmarks regarding different sequences in an exemplary dataset. **F,** *in vivo* imaging across scales of Jimt1 metastasis with Airyscan two-photon microscopy. **G,** Visualization of the voxel intensity extraction workflow of the co-registered MRI and 2P images.

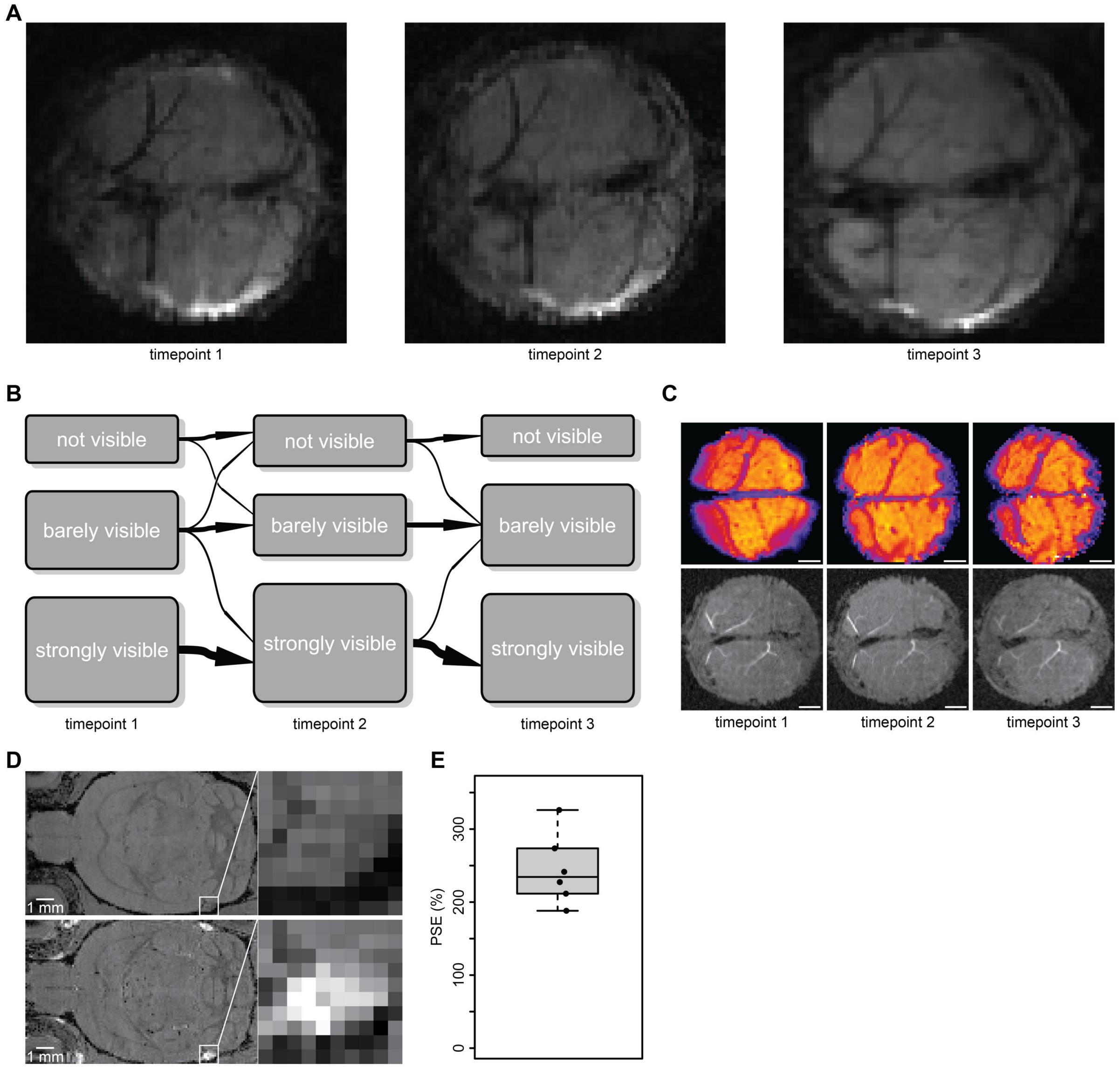

Figure S2 – Constant vessel visibility in MRI and equal contrast agent uptake in T1w sequence

A, T2w images of the same mouse at three different timepoints. Scale bars: 1 mm. **B,** Transition plot showing changes in vessel visibility in T2w images at three different timepoints (n = 68 vessels each measured at three timepoints, 18 T2w images of 6 mice). **C,** Upper row: T2* maps at three different timepoints indicating constant vessel visibility. Lower row: TOF images at three different timepoints indicating constant vessel visibility. Scale bars: 1 mm. **D,** Exemplary dataset of T1w image before (top) and after (bottom) injection of contrast agent in sigmoid sinus. Scale bars: 1 mm. **E,** Percent signal enhancement (PSE) of contrast agent uptake in sigmoid sinus (n = 6 datasets, each with one measurement before and one after injection of contrast agent).

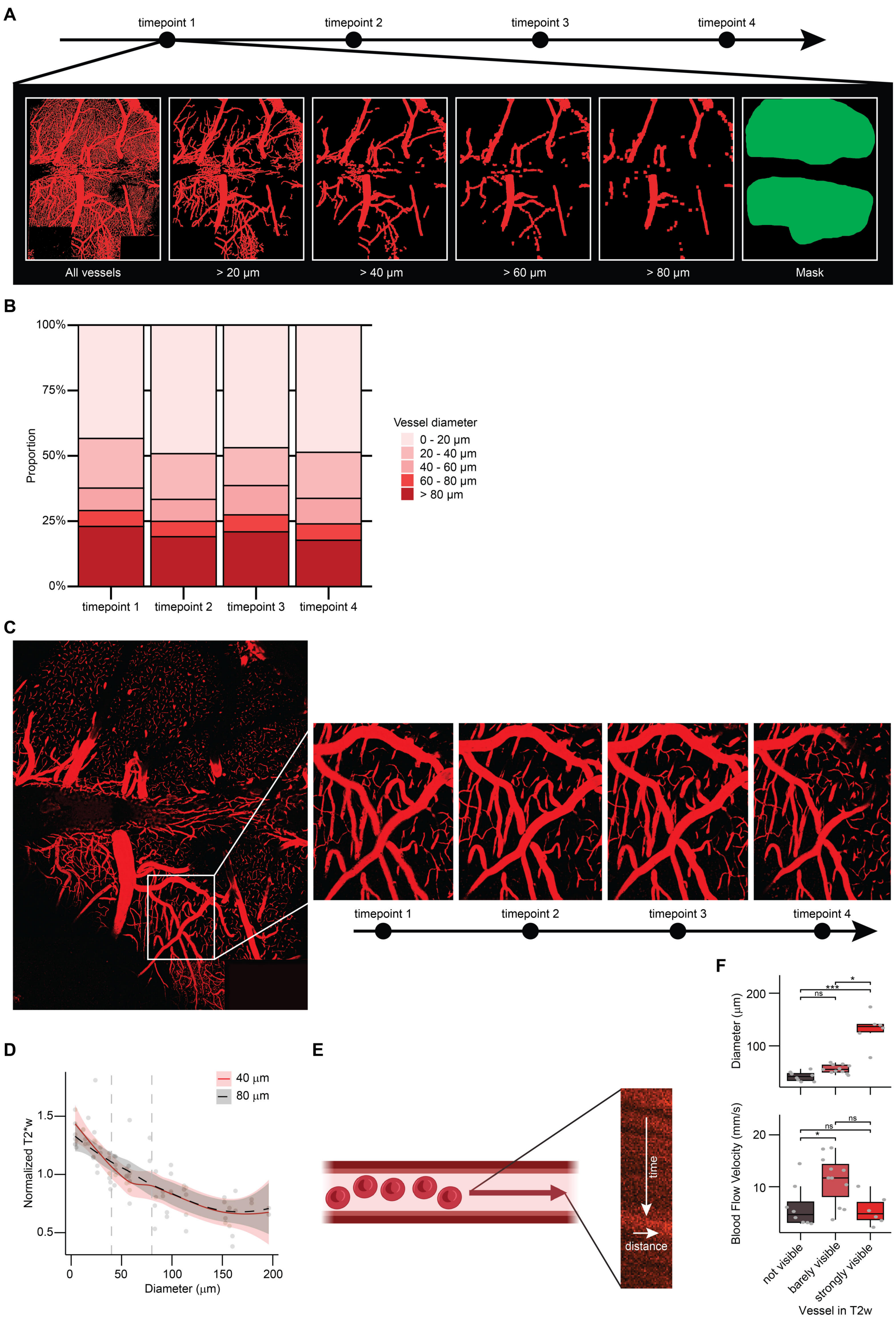

A
timepoint 1
timepoint 2
timepoint 3
timepoint 4
All vessels
> 20 µm
> 40 µm
> 60 µm
> 80 µm
Mask
B
100%
75%
50%
25%
0%
Proportion
timepoint 1
timepoint 2
timepoint 3
timepoint 4
Vessel diameter
0 - 20 µm
20 - 40 µm
40 - 60 µm
60 - 80 µm
> 80 µm
C
timepoint 1
timepoint 2
timepoint 3
timepoint 4
D
40 µm
80 µm
Normalized T2*w
1.5
1.0
0.5
0
50
100
150
200
Diameter (µm)
E
time
distance
F
Diameter (µm)
200
100
ns
***
*
Blood Flow Velocity (mm/s)
20
10
*
ns
ns
not visible
barely visible
strongly visible
Vessel in T2w

Figure S3 – Equal TRITC distribution to achieve consistent angiograms in two-photon microscopy

A, Visualization of filtered vessel diameters. **B,** Distribution of the volume of different vessel diameter categories in the same mouse at weekly intervals (n = 4 timepoints in 1 mouse). **C,** Illustration of visibility of small arterioles at four timepoints. Scale bar: 1 mm. **D,** Line plot with smoothing function showing comparison of vessel diameters against T2*w intensities between MRI measurements at 40 µm and 80 µm resolution. Dashed lines indicate 40 µm (left) and 80 µm (right) (n = 40 vessel pairs in 1 mouse). **E,** Schematic of intravital blood flow measurement. **F,** Top: Diameter of vessels depending on visibility in T2w. Bottom: Blood flow velocity in vessels depending on visibility in T2w. Median with interquartile range (n = 2 mice; for T2w: not visible, n = 8 vessels; barely visible, n = 11 vessels; visible, n = 2 vessels; strongly visible, n = 4 vessels; Kruskal-Wallis test followed by Dunn-Bonferroni post-hoc test).

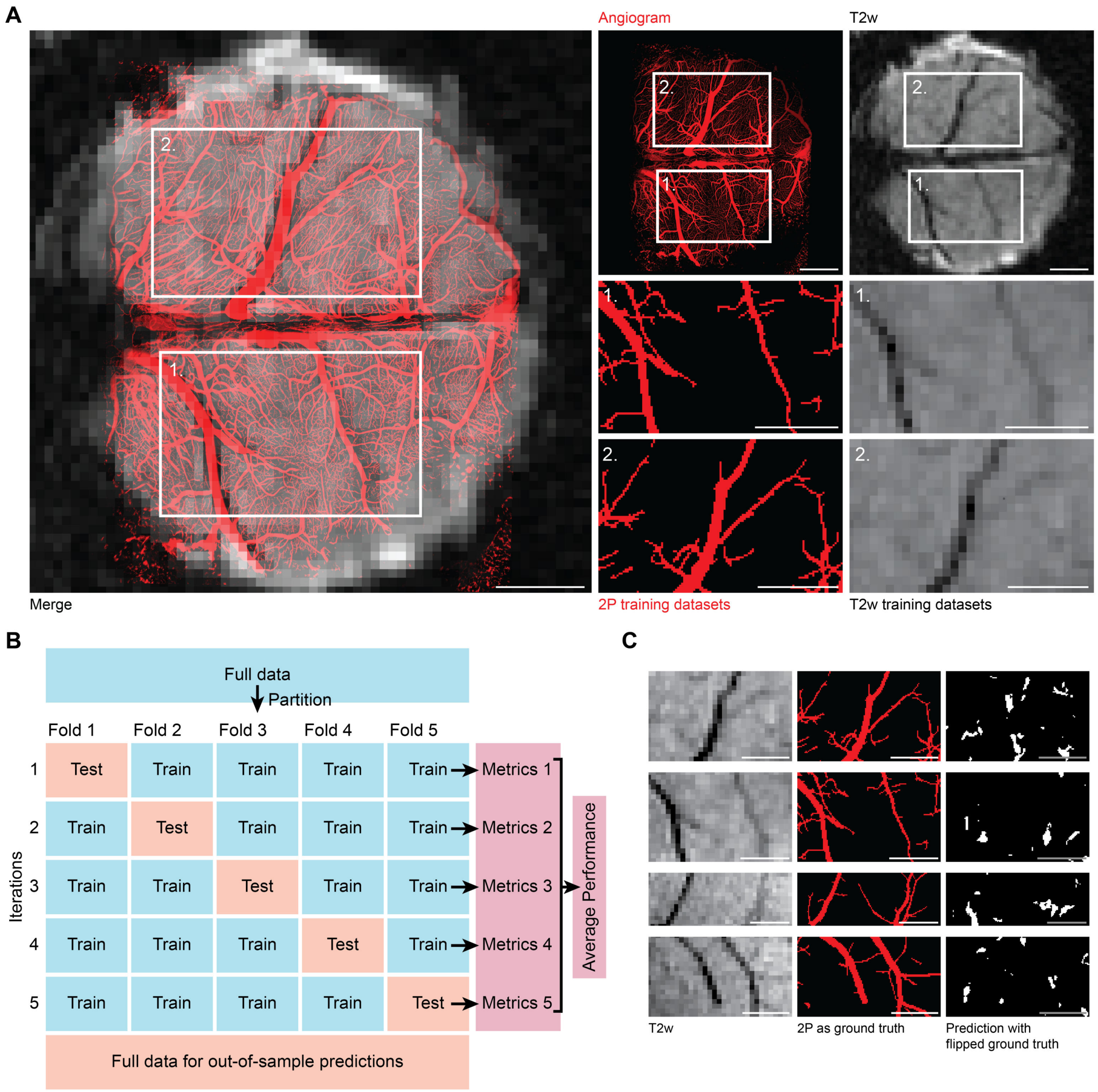

Figure S4 – BRIDGE simplifies automatic segmentation of MR images using microscopy as ground truth

A, Left: Merge of 2P angiogram and T2w image in control dataset. Middle: 2P angiogram and two training datasets created from the control dataset. Right: T2w image and two training datasets created from the control dataset. Scale bars: 1 mm. **B,** Illustration of 5-fold cross-validation. **C,** Left: T2w input crops. Middle: preprocessed 2P data as ground truth. Right: predictions of training with vertically and horizontally flipped 2P data as ground truth to confuse the convolutional neural network. Scale bars: 1 mm.

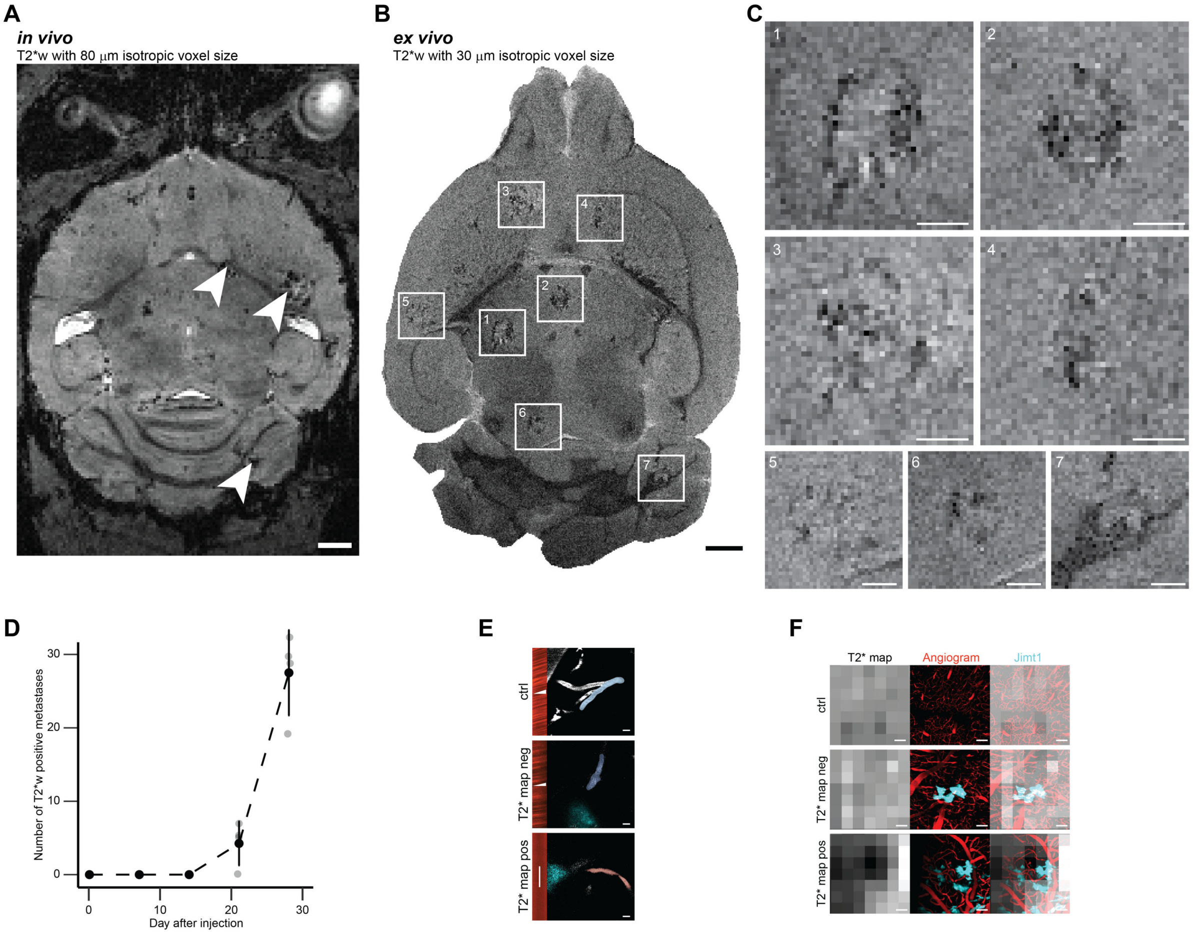

Figure S5 – Breast-cancer metastases show distinct T2*w positive lesions

A, *In vivo* T2*w sequence of Jimt1 breast cancer brain metastasis with isotropic voxel size of 80 µm. Arrowheads show metastases with T2*w hypointensities. Scale bar: 1 mm. **B,** *Ex vivo* high resolution T2*w sequence of Jimt1 breast cancer brain metastasis with isotropic voxel size of 30 µm. Squares show metastases with T2*w hypointensities. Scale bar: 1 mm. **C,** Larger illustrations of the outlined metastases from c. Scale bars: 300 µm. **D,** Temporal quantification of *in vivo* T2*w hypointense lesions in Jimt1 brains over 28 days (n = 4 mice, 20 datasets, 5 datasets per time point). **E,** Examples of blood flow velocity in control, T2*-negative lesion, and T2*-positive lesions. Scale bars: 10 µm. **F,** Examples of control, T2*-negative lesion, and T2*-positive lesions. Scale bars: 100 µm.

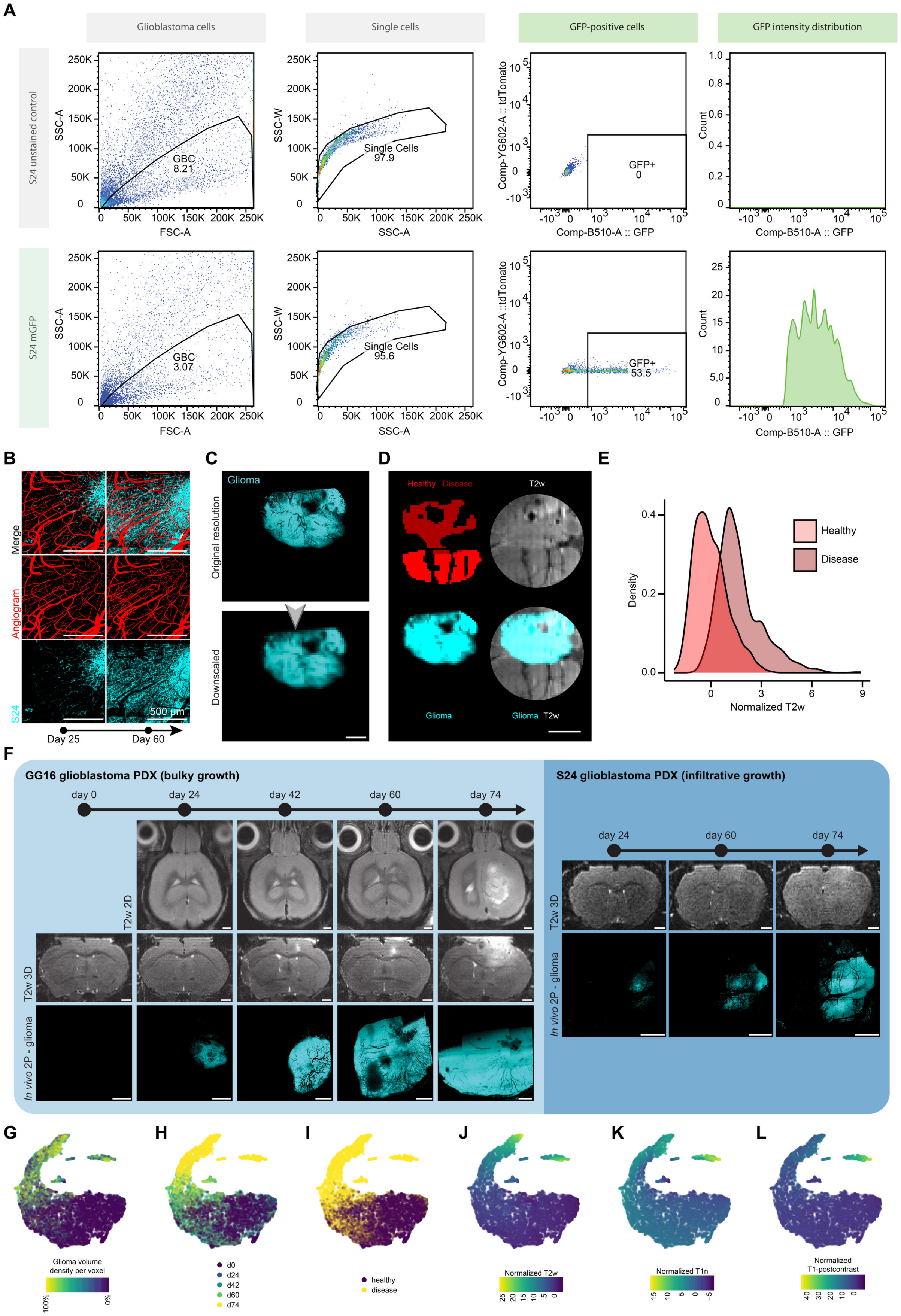
A
Glioblastoma cells
Single cells
GFP-positive cells
GFP intensity distribution
S24 unstained control
S24 mGFP
SSC-A
FSC-A
GBC
8.21
3.07
SSC-W
Single Cells
97.9
95.6
Comp-YG602-A :: tdTomato
Comp-B510-A :: GFP
GFP+
0
53.5
Count
B
Merge
Angiogram
S24
500 µm
Day 25
Day 60
C
Glioma
Original resolution
Downscaled
D
Healthy
Disease
T2w
Glioma
E
Healthy
Disease
Density
Normalized T2w
F
GG16 glioblastoma PDX (bulky growth)
S24 glioblastoma PDX (infiltrative growth)
day 0
day 24
day 42
day 60
day 74
T2w 2D
T2w 3D
In vivo 2P - glioma
G
H
I
J
K
L
Glioma volume density per voxel
100%
0%
d0
d24
d42
d60
d74
healthy
disease
Normalized T2w
Normalized T1n
Normalized T1-postcontrast

Figure S6 – Correlative signal intensity measurements in glioblastoma

A, Gating strategies exemplary for sorting S24 mGFP (green, n = 100000 cells) with FACS using unstained controls (gray, n = 51359 cells). **B,** Enlarged view of vessels and tumor on day 25 and day 60 after S24 glioma injection. Scale bars: 500 µm. **C,** Exemplary 2P image of GG16 with 2P resolution (1.18 µm) and downscaling to MR resolution (100 µm). Scale bars: 1 mm. **D,** 2P-MRI correlation of GG16 tumor (cyan) across multisequence MRI including T2, T1-native, and T1-postcontrast. Healthy tissue (red) and tumor (dark red). Scale bars: 1 mm. **E,** Density plot of normalized MR voxel grouped in “healthy” (red) and “disease” (dark red) of an exemplary dataset (day 42 after GG16 injection) (n = 1 dataset, 4007 voxels). **F,** Illustration of tumor growth in GG16 and S24 in MRI and 2P microscopy. Scale bars: 1 mm. **G-L,** UMAP dimension reduction of normalized T2w, T1-native, and T1-postcontrast data for GG16, labeled by glioma volume density (G), days after injection (H), mask categories (I), normalized T2w (J), normalized T1n (K) and normalized T1-postcontrast (L) (n = 4 mice, 13 datasets, 27555 voxels).

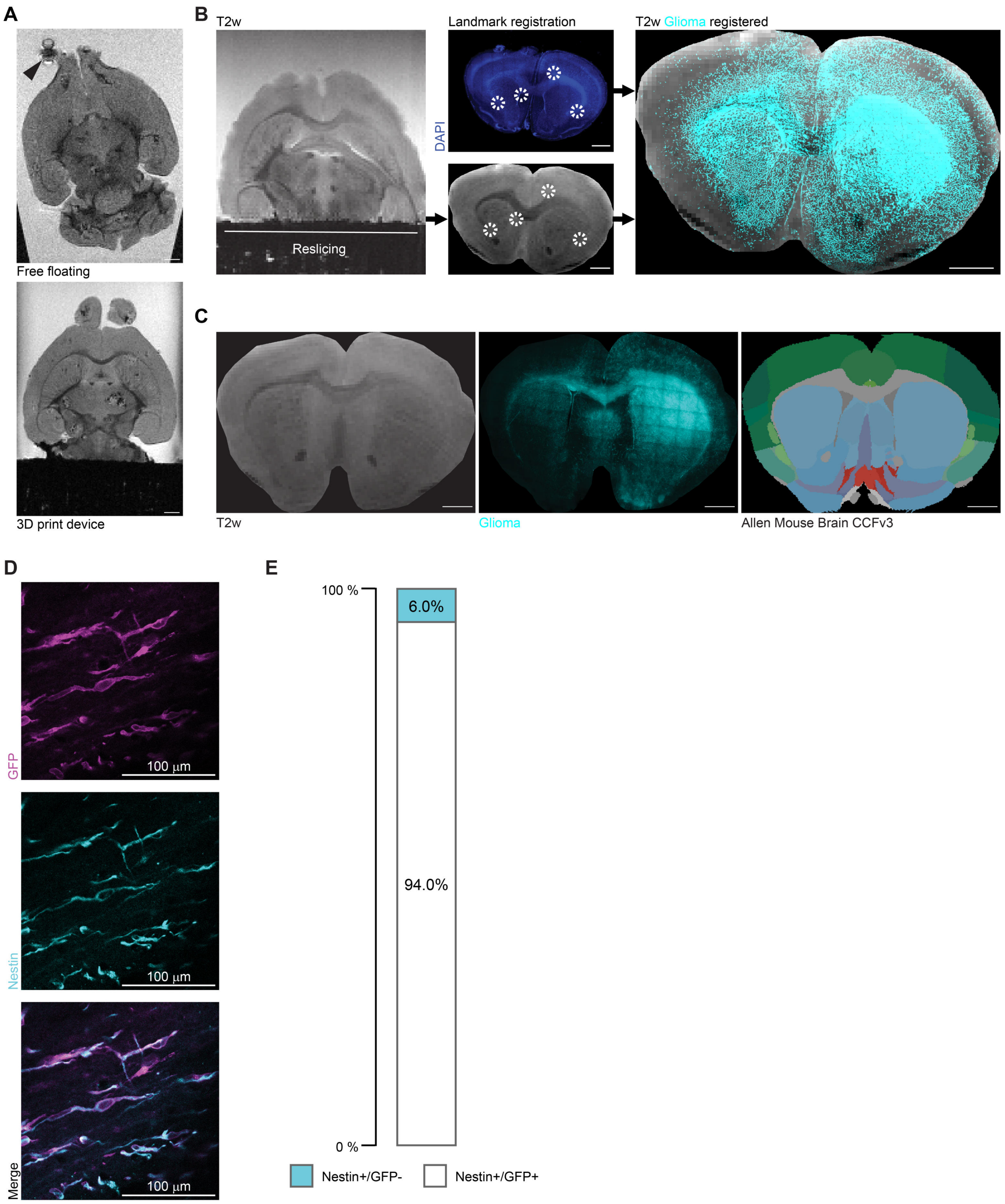

Figure S7 – Bespoke 3D-printed device for enhanced *ex vivo* brain co-registration

A, Comparative *ex vivo* T2*w imaging of Jimt1 xenograft mice brains acquired without (top) and with (bottom) 3D print device. Arrowhead depicts a gas bubble artifact. Scale bars: 1 mm. **B,** Computational integration of reslicing, MRI upscaling and 3D landmark registration in an S24 mGFP PDX slice. DAPI (blue) and Nestin (cyan). Scale bars: 1 mm. **C,** Slice alignment

with Allen Mouse Brain CCFv3. Scale bars: 1 mm. **D,** Confocal imaging of mouse brain slice of the S24 mGFP xenograft model with immunohistochemistry. GFP in magenta, Nestin in cyan. Scale bars: 100 µm. **E,** Stacked barplot of distribution of cells expressing Nestin and GFP (n = 67 cells).

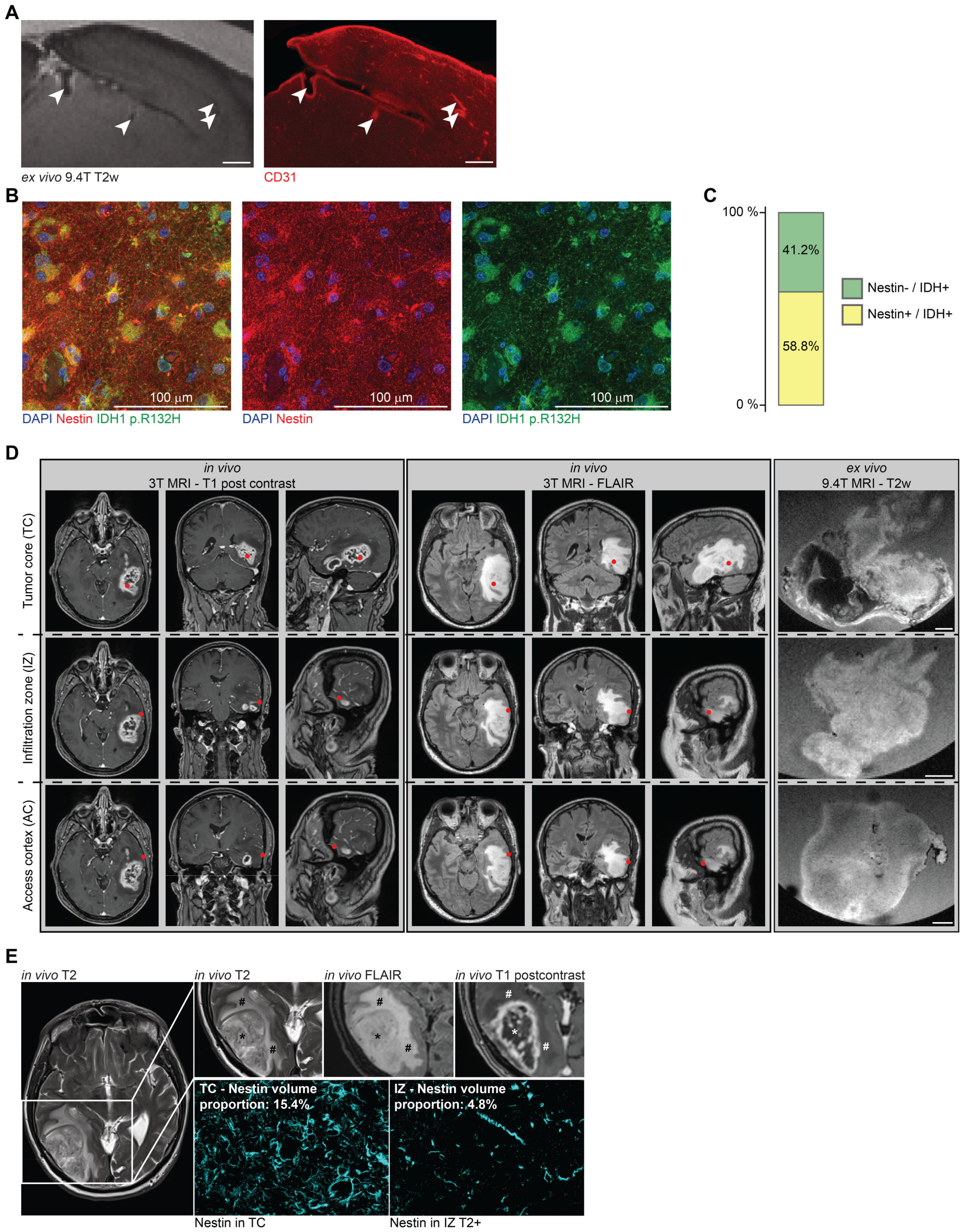

Figure S8 – Mapping of human glioma tissue to *ex vivo* MRI and clinical neuronavigated MRI

A, *Ex vivo* 9.4T T2w image as well as CD31 (red) acquisition. Arrowheads indicate vessels. Scale bars: 1 mm. **B,** Confocal image of human IDH mutant astrocytoma tissue. DAPI (blue),

IDH1 p.R132H (green), Nestin (red). Scale bars: 100 µm. **C,** Percentage of overlapping cells in the tissue shown in (B) (n = 17 cells). **D,** Mapping of *ex vivo* human tissue to clinical T1 post-contrast and FLAIR in one patient via neuronavigation. Scale bars: 1 mm. **E,** Left: Clinical T2w image of a patient with glioblastoma. Upper row shows clinical T2, FLAIR and T1 postcontrast images. TC indicates tumor core and IZ indicates infiltration zone. Stars indicate TC and hashtags indicate IZ. Lower row shows Nestin signal in TC with a Nestin volume proportion of 15.4% and in IZ with a Nestin volume proportion of 4.8% in the same patient.

Movie S1 – Longitudinality, multimodality and precision of the BRIDGE co-registration pipeline *in vivo*

Visualization of the different sequences on which 2P microscopy is registered based on unique vessel branches. Even small vessels show correspondence between MRI and 2P microscopy. Time course and tumor growth follow.

Movie S2 – Bridging scales from MRI to super-resolution microscopy *ex vivo*

The 3D representation of a brain mask segmented in MRI images is followed by a slice of the *ex vivo* T2w sequence. Microscopy was registered on this slice. Expansion microscopy was performed to see individual NGS.

Movie S3 – Clinical translation bridges scales from MRI to super-resolution microscopy in human glioma tissue

Neuronavigation mask is followed by the *ex vivo* T2w sequence. Microscopy was co-registered. In the following, expansion microscopy was performed to visualize glioma cells in 3D.

Table S1 – Tumor cell line classification

Table S2 – *In vivo* MRI sequences

Table S3 – *Ex vivo* MRI sequences

Table S4 – Reagents

Table S5 – Organisms and strains

Table S6 – Devices

Table S7 – Programs

Table S8 – Source data
